# Cross-Species Transmission Potential of H4 Avian Influenza Viruses in China: Epidemiological and Evolutionary Study

**DOI:** 10.3390/v16030353

**Published:** 2024-02-24

**Authors:** Shuxia Lin, Ye Zhang, Jiaying Yang, Lei Yang, Xiyan Li, Hong Bo, Jia Liu, Min Tan, Wenfei Zhu, Dayan Wang, Yuelong Shu

**Affiliations:** 1 School of Public Health (Shenzhen), Shenzhen Campus of Sun Yat-sen University, Shenzhen 518107, China; linshx29@mail2.sysu.edu.cn (S.L.);; 2 School of Public Health (Shenzhen), Sun Yat-sen University, Guangzhou 510006, China; 3 National Institute for Viral Disease Control and Prevention, Chinese Center for Disease Control and Prevention; WHO Collaborating Center for Reference and Research on Influenza, Key Laboratory for Medical Virology, National Health Commission, Beijing 102206, China; zhangye@cnic.org.cn (Y.Z.);; 4 Key Laboratory of Pathogen Infection Prevention and Control (MOE), State Key Laboratory of Respiratory Health and Multimorbidity, National Institute of Pathogen Biology, Chinese Academy of Medical Sciences & Peking Union Medical College, Beijing 102629, China

**Keywords:** H4, avian influenza virus, epidemiological, evolutionary, cross-species transmission, potential threat

## Abstract

H4 avian influenza viruses (AIVs) have been widely detected in live poultry markets in China. However, the potential public health impact of H4 AIVs remains largely uncertain. Here, we fully analyzed the distribution and phylogenetic relationship of H4 AIVs in China. We obtained 31 isolates of H4 viruses in China during 2009–2022 through surveillance in poultry-associated environments, such as live poultry markets and poultry farms. Genomic sequence analysis together with publicly available data revealed that frequent reassortment and introduction of H4 AIV from wild birds to poultry may have occurred. We identified 62 genotypes among 127 whole genome sequences of H4 viruses in China, indicating that H4 AIVs had great genetic diversity in China. We also investigated molecular markers and found that drug resistance mutations frequently occurred in the M2 protein and a few mutations related to receptor binding and the host signature in H4 AIVs. Our study demonstrates the cross-species transmission potential of H4 AIVs in China and provides some reference significance for its risk assessment.

## 1. Introduction

Avian influenza virus (AIV) belongs to influenza A virus, which is a member of the genus *Orthomyxovirus*. The genome of AIV consists of eight negative-sense single-stranded RNA segments that encode at least 10 viral proteins [1]. Based on antigenic differences between the surface glycoproteins hemagglutinin (HA) and neuraminidase (NA), 18 HA subtypes and 11 NA subtypes have been identified. Wild waterfowls are considered to be natural reservoirs for AIVs, encompassing all known subtypes except for H17N10 and H18N11, which were exclusively detected in bats [1,2,3,4].

AIV is a potential source for the emergence of human influenza pandemics. Historically, there have been at least four notable human pandemics related to AIVs: A(H1N1) in 1918, A(H2N2) in 1957, A(H3N2) in 1968, and swine-origin H1N1 in 2009 [5,6]. The emergence of these pandemic viruses were possibly triggered by the direct adaptation of AIVs in mammalian hosts or reassortment to acquire mammalian adaptations [7,8,9,10]. In addition to subtypes H1, H2, and H3, there are other subtypes, including H5, H6, H7, H9, and H10, that have been reported to spill over into humans and cause sporadic human infections [11,12]. It is questionable whether other subtypes could spill over, such as the H4 subtype which has been frequently detected in wild birds and poultry [13,14,15,16].

H4 AIVs were first isolated from a duck in Czechoslovakia in 1956 [17]. H4 AIVs are not only widely prevalent in wild and domestic bird species around the world [14,15,17,18,19,20], but they have also spilled over into mammals (e.g., seals and swine) [21,22,23]. At least five cases of H4 AIV infection in swine under natural conditions have been reported in Canada [24], the USA [21], and China [23,25]. Increasing seroepidemiologic evidence also demonstrated that H4 AIVs can infect pigs in China [26,27] and even poultry farmers and workers in the United States and Lebanon [28,29]. These instances point to the fact that H4 AIV has posed cross-species transmissibility, suggesting its potential for human infection.

In China, H4 AIVs were detected in domestic ducks as early as the 1980s, commonly the H4N6 subtype [30]. Recent studies found that H4 AIVs with various NA subtypes (such as N2, N3, N6, and N8) have been detected in live poultry markets (LPMs) in China [14,16,31,32]. They were widely isolated from domestic ducks and were sporadically detected from domestic geese and terrestrial domestic chicken [31,32]. Of note, H4 AIVs were frequent subtypes of co-infections with H3, H5, H6, H7N9, and H9N2 AIVs in LPMs of China, among which H3 was the most common [16,33]. A genetic study has revealed that H4 AIVs genome underwent complex reassortment with multiple subtypes of viruses [14]. Therefore, comprehensive systematic analyses of H4 AIVs in China are urgent.

In this study, we obtained 31 isolates of H4 viruses from poultry-related environments in China during 2009–2022. We fully analyzed the distribution and phylogenetic relationship of H4 AIVs in China to elucidate the epidemiological and genetic characteristics. Our study provides an important basis for the prevention and control of avian influenza.

## 2. Materials and Methods

### 2.1. Sample Collection

Between January 2009 and December 2022, environmental samples were collected from poultry-related environments (LPMs, poultry farms, backyards, slaughterhouses and poultry processing companies) monthly across 31 provinces, autonomous regions and municipalities in Chinese mainland according to the guideline on AIV surveillance of the Chinese Center for Disease Control and Prevention (China CDC). The environmental samples included poultry feces, sewage, poultry drinking water, surface swabs from poultry cages, etc. Feces or swab samples were maintained in a tube with 5 mL sampling solution. Liquid samples (such as sewage and poultry drinking water) were collected in a tube with 5–10 mL liquid. All samples were stored at 4 °C and sent to local Chinese National Influenza Surveillance Network laboratories within 48 h.

All samples were centrifuged at 3000 rpm for 10 min, and the supernatant was harvested for further testing. Influenza A viruses were identified by real-time reverse transcription PCR (real-time RT-PCR). Of these, positive samples of real-time RT-PCR were sent to the Chinese National Influenza Center (CNIC) for virus isolation.

### 2.2. Virus Isolation and Identification

Furthermore, 200 μL of the positive specimens, identified via real-time fluorescence quantitative PCR, were inoculated into 9- to 11-day-old specific-pathogen-free (SPF) embryonated chicken eggs. After incubation for 48–72 h at 37 °C and placement for 18 h at 4 °C, viruses were isolated from the allantoic fluids of eggs, and the presence of the virus was determined by a hemagglutinin (HA) assay using 1% turkey red blood cells (TRBCs). The subtypes of isolated viruses were identified via real-time RT-PCR and stored at −80 °C.

### 2.3. RNA Extraction and Genome Sequencing

Virus RNA was extracted from the isolated viruses using the MagMAX CORE Nucleic Acid Purification Kit (Thermo Fisher, Waltham, MA, USA). The extracted RNA was subjected to reverse transcription and amplification using the SuperScript^®^ III One-Step RT-PCR system (Thermo Fisher, Waltham, MA, USA) according to the described method [34]. The whole genome sequencing of influenza A virus was implemented on the automatic Applied Biosystems 3730xl DNA Analyzer (Life Technologies, Waltham, MA, USA) or the MiSeq high-throughput sequencing platform (Illumina, Inc., San Diego, CA, USA). The raw data from the MiSeq platform were paired reads with a length of 150 bp. Low-quality reads were trimmed, and the filtered reads were sampled and de novo-assembled using Velvet (version 1.2.10) [35] and Newbler (version 2.5). Contigs were blasted against a database containing all influenza A virus nucleotide sequences collected from the National Center for Biotechnology Information (NCBI) (http://www.ncbi.nlm.nih.gov/genomes/FLU, accessed on 26 March 2023) and the Global Initiative on Sharing All Influenza Data (GISAID) (http://www.gisaid.org, accessed on 26 March 2023). Sequences with the highest similarity were selected as references for mapping of reads using Bowtie 2 (version 2.1.0) [36]. The influenza A virus genome sequences were obtained by extracting the consensus sequences from the mapping results, with a coverage depth of at least 30 times at each site on the eight segments.

### 2.4. Sequence Collection and Collation

All sequences of 31 environmental H4 isolates were initially obtained in our surveillance, and one of them has been published in our previous study [37]. Sequences of all host-origin H4 HA genes and avian-origin N2, N3, N6, N8, and internal protein-coding genes were downloaded from the public databases, including the GISAID and NCBI, as of 26 March 2023. The sequences of public databases were collated and merged according to the following: (1) remove duplicate sequences in NCBI that have the same INSDC_Upload number as those in the GISAID; (2) for the same gene segment with multiple segment IDs in the GISAID, retain one of the segment IDs containing the full-length coding sequences (CDSs), otherwise retain the longer sequence; (3) for the sequences with the same isolate name between GISAID and NCBI, retain one of the sequences containing the full-length CDS, otherwise retain the longer sequence; and (4) remove the sequence if the strain name contains the keywords including “Recombinant”, “RG”, or “unpublished”.

### 2.5. Information Collation and Epidemiological Analysis

The metadata of H4 AIVs were collated according to the following: (1) the host origin was determined based on the isolate name, related reference titles, isolate information on the NCBI, and similar isolate names and categorized into poultry, poultry-related environment, wild birds, wild birds-related environment, mammals, and unknown; and (2) the location was determined based on the isolate name and classified into Europe, Oceania, Africa, North America, sub-Asia region and China.

In China, a total of 294 H4 AIVs, including mixed strains and those strains with an unknown NA subtype, collected during 1976–2022, were extracted. After excluding unknown host viruses, there were 277 (94.2%) H4 AIVs included in further epidemiological analysis.

### 2.6. Sequence Alignment and Phylogenetic Analysis

Due to a large number of sequences of NA and internal protein-coding genes, they were reduced using CD-HIT (version 4.8.1) at a cluster threshold ranging from 98.0% to 99.0% [38]. Sequences of all segments were aligned using MAFFT (version 7.490) and trimmed manually in MEGA (version 7.0) to obtain the CDS [39,40]. The signal peptide of the HA sequence was cut. Sequences with ≥95% CDS length and ≤3 ambiguous bases for all segments were included for phylogenetic analysis. The above datasets were used as a backbone for constructing the phylogenetic tree.

The gene sequences of all H4 AIV isolates from China in the public database were retained as representative sequences. To improve the credibility of the branching, we downloaded the sequences of avian-origin NA and internal protein-coding genes from the GISAID to create a local database. Using the basic local alignment search tool (BLAST), we retrieved 20 sequences that shared the highest nucleotide similarity for each representative sequence of NA and internal protein-coding genes in the local database [41].

Maximum likelihood (ML) phylogenies of all segments were reconstructed using the FastTree (version 2.1.11) under the GTRGAMMA model with 1000 bootstrap replicates [42]. To ensure accuracy, only one sequence was retained from the same host, time, and location. To improve the visualization of the phylogenetic tree, some sequences were manually removed, ensuring all sequences closely related to the H4 avian influenza virus in China. The resulting trees were classified into divergent lineages or sublineages based on the topology of phylogenetic trees with bootstrap support values ≥ 70%. The FigTree (version 1.4.4) (http://tree.bio.ed.ac.uk/software/figtree) and iTOL (version 6) (https://itol.embl.de) were used for visualization and annotation.

### 2.7. Genotypic Analysis

Frequent reassortment occurred in AIVs, resulting in a wild bird gene pool. AIVs maintained in wild birds spilled over into novel hosts, such as domestic poultry and mammals, leading to the emergence of lineages/sublineages. For strains with all eight gene segments available, genotypes were determined by combinations of lineage/sublineage assignments of each segment. Moreover, 127 H4 AIV strains isolated from China between 1976 and 2022, involving subtypes H4N2, H4N3, H4N6, and H4N8, were carried out for the genotypic analysis. For each NA subtype, genotypes were named G1 to Gn, according to the time of identification.

## 3. Results

### 3.1. H4 AIVs in China

**Figure 1:**
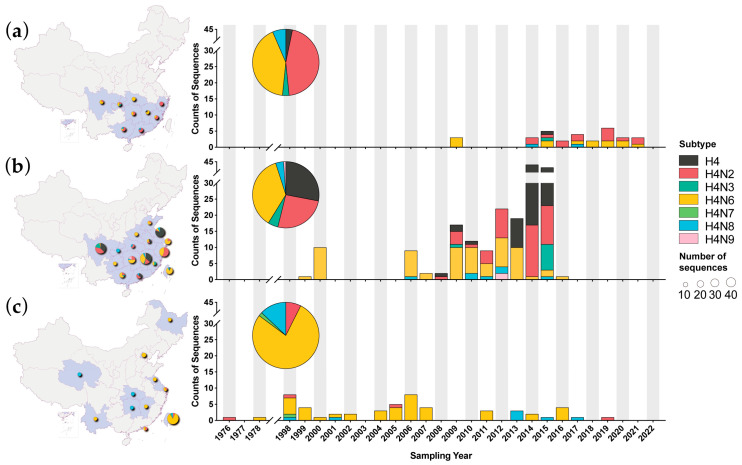
Spatiotemporal distribution of H4 AIVs with known avian host sources in China during 1976–2022. (**a**) The distribution of H4 AIVs collected from poultry-related environments in our surveillance; (**b**) the distribution of H4 AIVs collected from poultry and poultry-related environments in the public databases; (**c**) the distribution of H4 AIVs collected from wild birds in the public databases. The **left** panel shows the geographic distribution of the H4 AIVs, and the size of the circles indicates the number of reported H4 AIVs; the **right** panel shows the temporal distribution of H4 AIVs with various subtypes.

Between 1 January 2009 and 31 December 2022, we isolated 31 H4 AIVs sporadically from nine regions in southern provinces in mainland China, with subtypes involving 14 H4N2, 13 H4N6, 2 H4N8, 1 H4N3, and 1 H4 mixed strain (Figure 1a, Table S1). Furthermore, two to six strains were isolated each year, except from 2010 to 2013 and 2022 when no strains were isolated. The most common subtypes, H4N2 and H4N6, were detected in seven years. The samples of these H4 viruses were from various sites, including twenty-one in live poultry markets, two in poultry farms, one in poultry processing companies, and seven in unknown sites (Table S1). The samples involved different types, including three of feces, three of drinking water, two of surface swabs of cages, two of sewage and twenty-one of others (Table S1). Since all samples were obtained from the environments, they could not be linked to a specific host.

In the public database, H4 AIVs from poultry were transiently reported in 1999 and 2000, followed by an absence of H4 AIV reports between 2001 and 2005 (Figure 1b). Since 2006, there has been an increasing trend in the detection of H4 AIVs in poultry. Especially during 2009–2015, there was a substantial number of sequences, accounting for 62.1% (172/277). In 2016, the number of sequences sharply declined, and no sequence was reported thereafter. In wild birds, after detections in 1976 and 1978, there was a gap in H4 AIV detection until 1998 (Figure 1c). Then, H4 viruses were intermittently detected in wild birds with a small number of and occasional interruptions in several years.

Despite discrepancies in the time intervals between our surveillance and public data, H4N2 and H4N6 were the main subtypes in poultry and poultry-related environments. H4N3, H4N8, and H4N9 were also detected. That was different among wild birds, where the H4N6 subtype was dominant, followed by H4N8, H4N2, and H4N7. Furthermore, one H4N1 AIV with unclear host information could not be classified as poultry or wild birds. H4N4 and H4N5 subtypes were not detected in birds in China. (Figure 1). Geographically, H4 AIVs from wild birds were scattered in 11 regions across China (Figure 1c), while H4 AIVs from poultry were concentrated in the southern provinces and two adjacent northern provinces (Shandong and Henan provinces) (Figure 1b).

### 3.2. Phylogenetic Analysis of HA Gene

**Figure 2:**
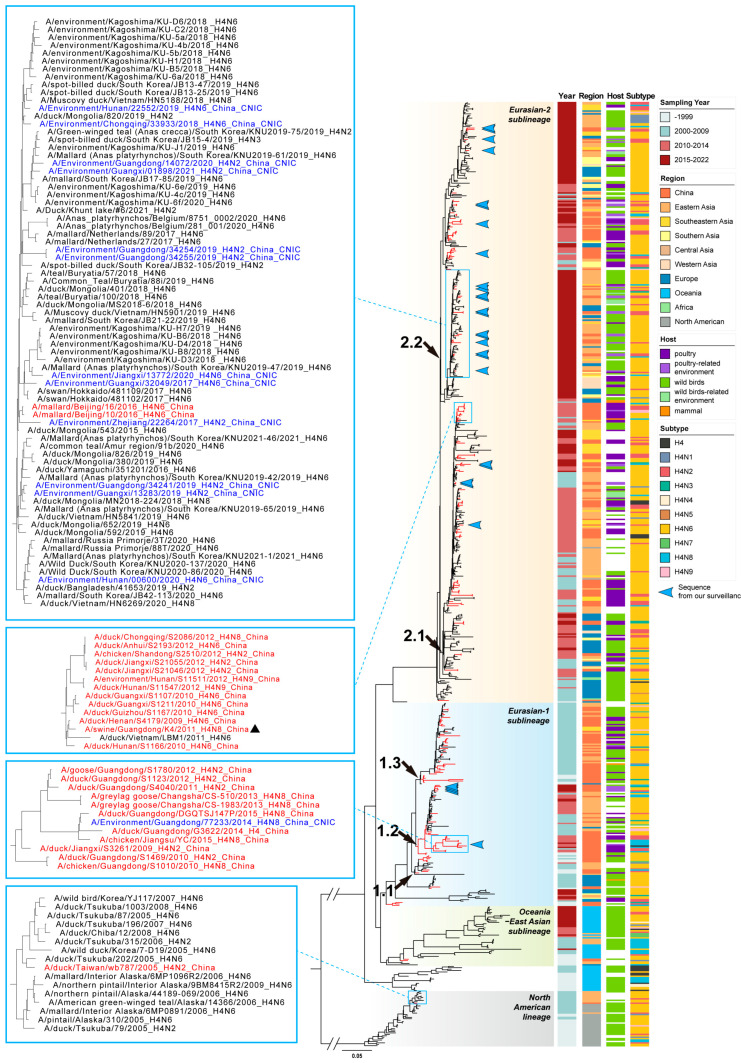
Maximum likelihood phylogenetic tree of HA gene of H4 AIVs (n = 668). Sequences were collected from public databases (GISAID and NCBI) and our surveillance. The red branches indicate H4 sequences from China; the blue arrows indicate sequences reported from our surveillance. Lineages and sublineages are shown with different background colors on the phylogenetic tree, and groups in sublineages Eurasian-1 and Eurasian-2 are marked with black arrows at the nodes. The sampling year, region, host sources, and subtype of sequences are annotated with colored bars shown on the right panel. Representative branches are indicated in boxes and magnified on the left panel; H4 strains sequenced from our surveillance are in blue; other H4 AIVs from China are in red; the swine-origin virus is marked with triangles. The scale bar indicates nucleotide substitutions per site.

To clarify the evolution of the HA gene of H4 AIVs in China, we performed a phylogenetic analysis using sequences from this study and reference sequences in public databases (GISAID and NCBI). Consistent with the previous study, the maximum likelihood tree of the HA gene was divided into the Eurasian lineage and the North American lineage (Figure 2 and Figure S1) [17]. Based on the topology of the tree and geographic distributions, the Eurasian lineage could be further classified into four sublineages: Eurasian-1, Eurasian-2, Oceania–East Asian, and Oceania, respectively. Except for one early HA gene in the North American lineage, all H4 AIV’s HA sequences in China belonged to the Eurasian lineage, with the majority in Eurasian-1 and Eurasian-2 sublineages. The Eurasian-1 sublineage could be further divided into clades 1.1, 1.2, and 1.3. The Eurasian-2 sublineage could be further divided into clades 2.1 and 2.2. These clades contained almost all H4 AIVs in China, among which clade 2.2 contained the majority of recent viruses.

In each clade, the H4 AIVs could be found throughout the Eurasian continent, with East Asia, including China, being the most active region. The viruses, isolated from poultry, poultry-related environments, wild birds and wild birds-related environments, were interlaced in a phylogenetic tree (Figure 2). Each lineage, sublineage, or clade had various NA combinations, with the H4N6 subtype being dominant.

The H4 AIVs from our surveillance of poultry-related environments were scattered in clades 1.2, 2.1, and 2.2 (Figure 2). These viruses were closely related to the H4 AIVs of poultry or wild birds from South Korea, Japan, Mongolia, and Vietnam, as well as to Chinese AIVs from other studies. Some small clusters of H4 AIVs in China were formed but did not maintain for a long time. For example, a cluster contained viruses that were identified in poultry, poultry-related environments, and even swine between 2009 and 2012 (Figure 2). Another cluster containing viruses from poultry, poultry-related environments, and wild birds circulated between 2009 and 2015 (Figure 2). There was one duck virus from Taiwan province located in the North American lineage, which grouped at a small branch with viruses from Japan and South Korea (Figure 2).

### 3.3. Phylogenetic Analysis of NA Genes

**Figure 3:**
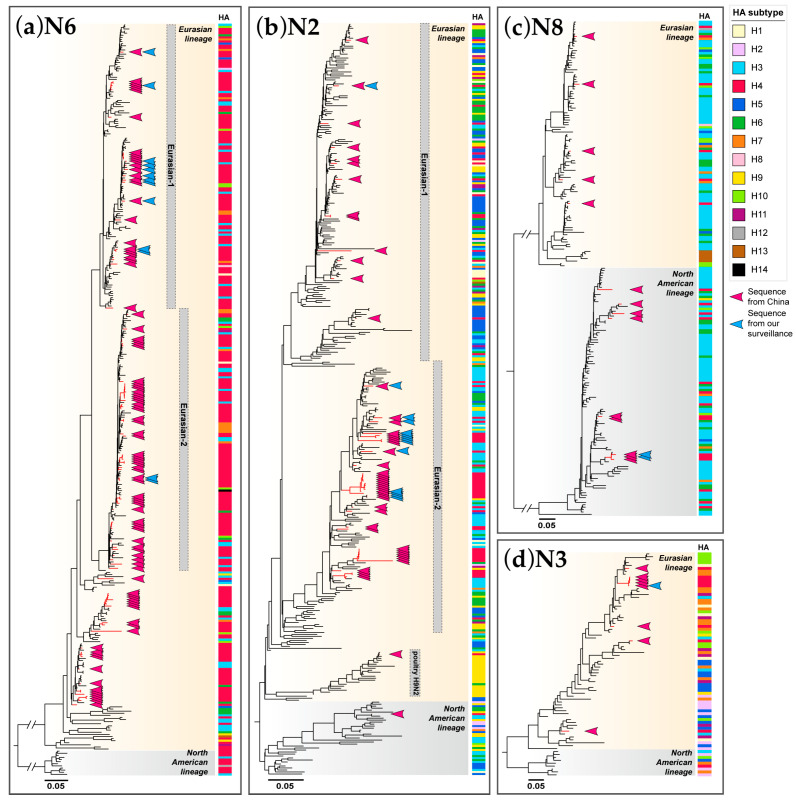
Maximum likelihood phylogenetic tree of NA gene. (**a**) N6 gene (n = 386); (**b**) N2 gene (n = 385); (**c**) N8 gene (n = 213); (**d**) N3 gene (n = 77). Sequences were collected from public databases (GISAID and NCBI) and our surveillance. Lineages are shown with different background colors on the phylogenetic tree; sublineages are labeled with gray wide vertical lines on the right. The red branches and red arrows indicate sequences from China; the blue arrows indicate sequences reported from our surveillance. The HA subtypes of sequences are annotated with colored bars shown on the right of the phylogenetic tree. The scale bar indicates nucleotide substitutions per site.

As various combinations of NA subtypes of H4 AIVs have been detected in China, we performed phylogenetic analyses of four major NA subtypes: N2, N3, N6, and N8. Each of these NA subtypes has evolved into two main lineages, Eurasian lineage and North American lineage. For each subtype, the NA genes of H4 AIVs formed a paraphyletic clade that mixed with NA genes from other HA subtypes. Almost all of the NA genes for H4 AIVs from China belonged to the Eurasian lineage, except for the NA genes of one H4N2 and 10 H4N8 AIVs located in the North American lineage (Figure 3b,c and Figure S2a,d).

Of the H4 AIVs, N6 was the most abundant NA subtype. All of the NA genes of H4N6 AIVs in China were scattered in the Eurasian lineage (Figure 3a and Figure S2c). Two sublineages, named Eurasian-1 and Eurasian-2, were divided from the Eurasian lineage. Most of the H4N6 viruses (11/13) in this study were scattered in the Eurasian-1 sublineage, while two viruses clustered into the Eurasian-2 sublineage. This differed from public data in China, which showed that more viruses were dispersed in the Eurasian-2 sublineage.

The Eurasian lineage of the N2 subtype also could be divided into sublineages, such as Eurasian-1, Eurasian-2, and H9N2 poultry sublineages (Figure 3b and Figure S2a). Most of the H4N2 viruses (13/14) in this study were scattered in the Eurasian-2 sublineage, while one virus clustered into the Eurasian-1 sublineage. This was comparable to Chinese H4N2 viruses, except that one Chinese H4N2 AIV clustered into the H9N2 poultry sublineage.

The NA genes of H4N8 AIVs from China were scattered in both Eurasian and North American lineages (Figure 3c and Figure S2d). We have isolated two H4N8 viruses that belonged to the North American lineage. All H4N3 AIVs from China contained NA genes from the Eurasian lineage including one from our surveillance (Figure 3d and Figure S2b).

### 3.4. Phylogenetic Analysis of Internal Protein-Coding Genes

We have reconstructed the phylogenetic analyses for all six internal protein-coding genes including all available sequences of H4 AIVs in China. Almost all genes of H4 AIVs in China fell into the Eurasian lineage, except for one PB1 and two MP sequences belonging to the North American lineage. For the specific concern and host, we further classified the internal protein-coding genes into the Eurasian wild bird gene pool (allele A and B only for NS gene), the ZJ-5 sublineage, the HPAI H5N1 and/or HPAI H5Nx sublineage, and the poultry H9N2 ZJ-HJ/07 sublineage (Figure S3a–f). Except for the MP gene of A/Environment/Chongqing/45279/2015(H4N3) being located in the North American lineage, all internal protein-coding genes of viruses isolated in our poultry-related surveillance were discretely distributed in the Eurasian lineage, with the majority in the Eurasian wild bird gene pool or a minority in the ZJ-5 sublineage. Other H4 AIVs from China comprised similar gene distributions, and very few sequences fell into the H9N2 or HPAI H5 sublineage.

### 3.5. Genotypic Analysis

**Figure 4:**
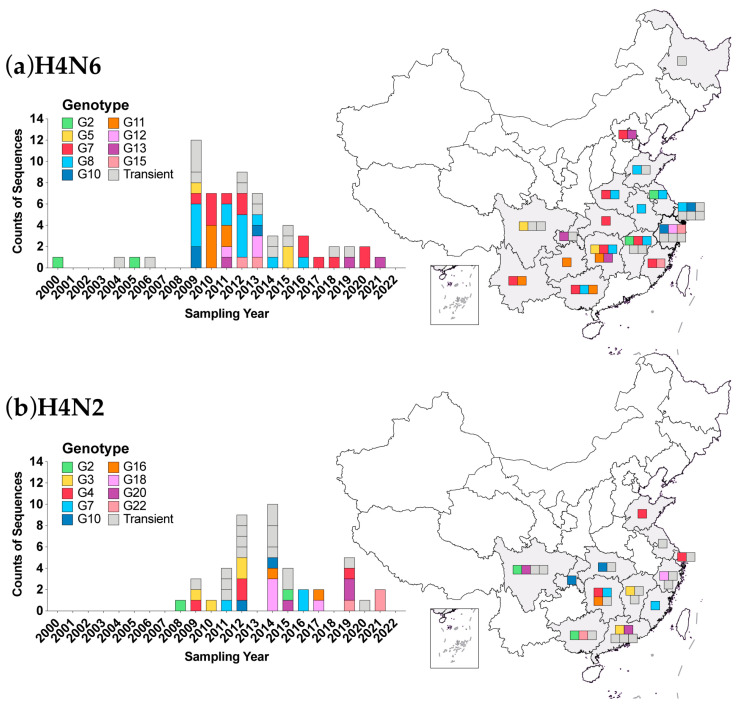
Diversity of genotypes of H4N6 and H4N2 AIVs in China during 2000–2022. (**a**) The distribution of genotypes of H4N6; (**b**) the distribution of genotypes of H4N2. The **left** panel shows the temporal distribution of the genotypes. The **right** panel shows the geographic distribution of the genotypes; provinces with detected genotypes are filled with gray color on the map; different genotypes are marked with square symbols of corresponding color on the map; transient genotypes are marked with gray square symbols.

The phylogenetic analysis showed that H4 AIVs in China had great genetic diversity, and frequent reassortment might occur with other AIVs. Based on the lineage/sublineage and gene pool classification of eight gene segments, we identified 62 genotypes among 127 whole genome sequences of H4 viruses in China, including twenty-four genotypes (G1-G24) from 45 H4N2 viruses, twenty-four genotypes (G1-G24) from 65 H4N6 viruses, ten genotypes (G1-G10) from 12 H4N8 and four genotypes (G1-G4) from five H4N3 viruses (Table S2). Most of the genotypes (42/62) were transiently detected in a year (Figure 4 and Figure S4). Some genotypes have been sporadically detected in 2–3 years and commonly in different provinces, including seven genotypes (G2, G5, G10, G11, G12, G13, and G15) for H4N6, nine genotypes (G2, G3, G4, G7, G10, G16, G18, G20, and G22) for H4N2, and two genotypes (G2 and G3) for H4N8 (Figure 4 and Figure S4a).

Only two genotypes, G7 and G8 of the H4N6 virus, have been continuously detected. The H4N6 G7 and G8 genotypes comprised the HA gene from the Eurasian-2 sublineage, all six internal protein-coding genes from the Eurasian wild bird gene pool, and the NA gene from Eurasian-1 and Eurasian-2 sublineages, respectively (Table S2). H4N6 G7 has been detected for 12 years in eight provinces (Figure 4a). H4N6 G8 has been detected for 8 years in eight provinces (Figure 4a).

Of note, the transient genotype H4N2 G14 contained NA, PB2, PB1, PA, NP, and NS genes from the poultry H9N2 sublineage. Another transient genotype H4N8 G6 contained the PB2 gene from the poultry H9N2 sublineage. Genotype H4N2 G9 had a whole set of internal protein-coding genes shared with HPAI H5 AIV. Genotype H4N6 G22 also shared the NS gene with HPAI H5 AIV (Table S2).

### 3.6. Molecular Characteristics

We further investigated the molecular markers possessed by 127 strains of the above genotypes. All HA genes had a single basic amino acid at the HA cleavage site (HACS) between HA1 and HA2, indicating that these strains were low-pathogenic avian influenza viruses (LPAIV). The amino acids (Q226 and G228, H3 numbering) at the receptor binding sites of HA genes were highly conserved in all H4 AIVs, suggesting that they retained the α2,3-linked sialic acid (avian-like) receptor binding preference [43,44]. However, one isolate of A/duck/Shanghai/421-2/2009 possessed mutation V214I, which might increase affinity for the α2,6-linked sialic acid (human-like) receptor in some genetic backgrounds (Table S3) [45]. There were five potential glycosylation motifs conserved in the HA protein of 96.1% (122/127) viruses at positions 2 NYT, 18 NGT, 162 NLT, 294 NIS, and 481 NGT (Table S4). For the remaining four strains, two lost glycosylation at 2 NYT, one lost at 18 NGT, and one added at 438 NDS were observed.

Mutations associated with reduced drug sensitivity in NA genes were not identified, such as E119V, H274Y, and R292K (N2 numbering) [46]. One isolate from H4N2 G14 possessed a 3-amino acids deletion at positions 63 to 65 in the NA stalk regions. There were no other deletions in the NA genes of any of the other viruses. No drug resistance mutation was found in the PA gene. In the M2 protein, fifteen H4 viruses possessed 27I/A, in which one virus also possessed 26F, and three viruses contained 31N (Table S3) [47,48]. In sum, 14.2% (18/127) strains contained mutations associated with increasing resistance to amantadine and rimantadine.

The mutations Q591K, E627K and D701N, which play an important role in influenza virus virulence and transmission in mammals, were not found in the PB2 gene [49,50,51]. One strain from H4N2 G4 had residue 482R that might be associated with increasing virus polymerase activity and virulence in mice [52]. Host signature amino acids were found in a few H4 AIVs, including PB2-9N, PB2-64T, PB2-105M, PB2-702R, PB1-327K, PA-57Q, PA-409N, NP-33I, and NP-455E, which might be related to a host specificity shift (Table S3) [53,54,55]. A 13-amino acids deletion at positions 218 to 230 in NS1 protein was found in an H4N2 G14 strain.

## 4. Discussion

AIVs in wild waterfowl are known as the source of influenza virus in other species. Multiple subtypes of AIV have continued to cross the species barrier and attempt to establish lineages/sublineages in the new host. In this study, we characterized the activity and genetic evolution of H4 AIVs in China, which is a hot spot for AIVs. The epidemiology analysis based on public databases suggested that H4 AIVs had a long-term circulation in wild and domestic birds across China. During 2006–2015, the number of sequences of H4 AIV has been increasing, which may be due to the enhanced surveillance of AIVs for the H7N9 outbreak [56,57,58]. We isolated 31 H4 viruses from poultry-relative environments between 2009 and 2022, which adequately replenished the gap in public databases where no sequences in poultry were available after 2017 (Figure 1a,b). Our results suggest that H4 AIVs circulated detectably in LPMs and poultry farms in China, although our samples were obtained from poultry-related environments and could not be associated with a specific host. However, according to public databases, it was shown that H4 AIVs in China were predominantly found in ducks, followed by chickens, geese, and quails.

Avian influenza viruses have been disseminated widely through the migration of wild aquatic birds and introduced into domestic poultry [59,60]. The HA genes of H4 AIVs in China were scattered in a phylogenetic tree and were close to those from other countries (Figure 2). Although a few small clusters were found in poultry, in most cases, H4 AIVs in wild birds and poultry were mixed on phylogeny. It implied that H4 AIV has not been established in the Chinese domestic bird population but is maintained by wild birds. The frequent introduction of H4 AIV from wild birds to poultry may have occurred. The long-term evolution of wild birds has given rise to sublineages and clades, of which clades 1.2, 2.1, and 2.2 contained most of the recent H4 AIVs circulating in China.

Reassortment is very common for H4 AIVs. Exchanging NA and internal protein-coding genes with other AIVs generated various viral subtypes and genotypes. H4N6 was the most abundant subtype in wild birds among H4 AIVs. In poultry, the proportion of the H4N2 subtype increased and was comparable to H4N6 in China (Figure 1). This suggested that the H4N2 subtype might have an advantage in poultry, which requires further investigation. Our results revealed that at least 62 genotypes of the H4 AIVs have been identified in China, while most of them were transiently detected. The vast majority of genotypes carried the internal protein-coding genes belonging to the Eurasian wild bird gene pool or the ZJ-5 sublineage which consisted mainly of viruses isolated from domestic waterfowl in China [61]. More genotypes were found in poultry and related environments than in wild birds (Table S5), which may be attributed to the much greater sequences available in poultry, and more reassortment could occur after introduction into poultry for host adaption. It is noteworthy that strains from poultry representing two genotypes obtained gene segments from poultry H9N2 AIVs.

Adaptive mutations known as molecular markers emerged in strains of some H4 genotypes that may contribute to changing the virus’ biological characteristics, e.g., host range, virulence and drug resistance. The anti-drug mutations more frequently happened to M2 inhibitors but not to NA or PA inhibitors, so drug stockpiles to response could be considerable. The receptor binding and host signature mutations were rare in H4 AIVs, but their effect on viral function needs to be verified by further experiments.

Undoubtedly, H4 AIVs have a potential risk of spillover infection to humans. First, they could circulate in live poultry markets, where they have been considered a major source of AIV dissemination, reassortment, and interspecies transfer [62,63]. Second, H4 AIVs were geographically widespread, having been detected from wild birds dispersed throughout China while being detected from poultry and related environments distributed in almost all provinces in southern China. Third, H4 AIVs were able to acquire gene segments derived from other AIVs, especially poultry viruses, and gained mutations associated with host signature and drug resistance. Fourth, mammalian infections have already occurred, with two cases of H4 AIVs in swine reported and seroepidemiologic evidence found in some swine populations in China [23,25,26]. Finally, it has been experimentally confirmed that H4 AIVs of duck origin can infect mice directly without prior adaptation [14,64], possess human-type receptor binding specificity, and transmit between guinea pigs via direct contact [14].

It is unpredictable which one will be the next pandemic virus. Here, we showed the potential of H4 AIVs in China and provided some reference significance for its risk assessment. There are still surveillance gaps for AIVs. Considering the pandemic potential of H4 AIVs, due attention should be paid to strengthening the surveillance of birds and mammals, sharing data timely, as well as conducting further investigation.

## 5. Conclusions

In conclusion, our study found that H4 AIVs circulated detectably in live poultry markets and poultry farms in China, mainly the H4N6 and H4N2 subtypes. The viruses possessed great genetic diversity due to frequent reassortment with other AIVs from both wild birds and poultry. The introduction of H4 AIV from wild birds to poultry may have occurred, but poultry well-adapted lineage still has not been established. Moreover, a few H4 strains showed mammalian adaptive mutations, which we should pay attention to, and we should further investigate their potential for cross-host transmission. Hence, it is crucial to enhance the surveillance of H4 AIVs.

## Data Availability

The data that support the findings of this study are available from the corresponding authors upon request.

## Supplementary Materials

The following supporting information can be downloaded at: https://www.mdpi.com/article/10.3390/v16030353/s1, Figure S1: Phylogenetic tree of H4 hemagglutinin (HA) genes; Figure S2: Phylogenetic tree of neuraminidase (NA) genes; Figure S3: Maximum likelihood trees of internal protein-coding genes; Figure S4: Diversity of genotypes of H4N8 and H4N3 AIVs in China during 2000–2022; Table S1: Detailed information of 31 environmental H4 subtype viruses obtained in our surveillance; Table S2: Genotypes of all H4 subtype AIVs with the whole genome; Table S3: List of H4 AIVs and genotypes with key molecular markers in China; Table S4: Potential glycosylation motifs of HA protein.; Table S5: Genotypes of H4N2, H4N3, H4N6 and H4N8 from wild birds, poultry, and unknown.
