# Supplementary material for: Cross-Species Transmission Potential of H4 Avian Influenza Viruses in China: Epidemiological and Evolutionary Study
Source: Viruses. 2024 Feb 24;16(3):353. doi: 10.3390/v16030353 (PMC10974465; doi:10.3390/v16030353)
Supplement: Supplementary file 1 [file viruses-16-00353-s001.zip › Supplementary Figure S1.pdf]

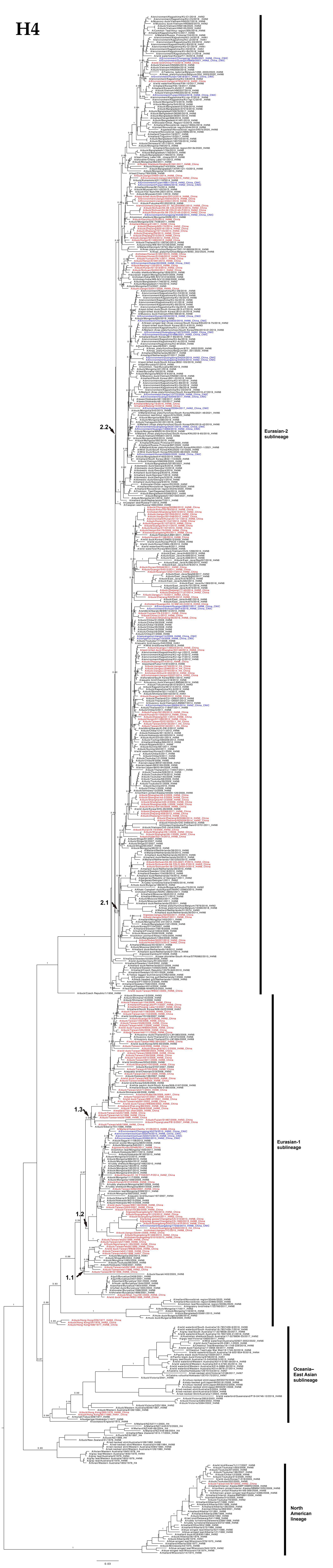

Figure S1. Phylogenetic tree of H4 hemagglutinin (HA) genes. Maximum likelihood tree (n=668). H4 strains sequenced in this study are in steel blue. Other H4 AIVs in China are in red. Branch lengths are scaled according to the number of substitutions per site. Branch support values of selected nodes are shown. Sublineages and subgroups are labeled with vertical lines on the right.
