# Supplementary material for: Cross-Species Transmission Potential of H4 Avian Influenza Viruses in China: Epidemiological and Evolutionary Study
Source: Viruses. 2024 Feb 24;16(3):353. doi: 10.3390/v16030353 (PMC10974465; doi:10.3390/v16030353)
Supplement: Supplementary file 1 [file viruses-16-00353-s001.zip › Supplementary Figure S2.pdf]

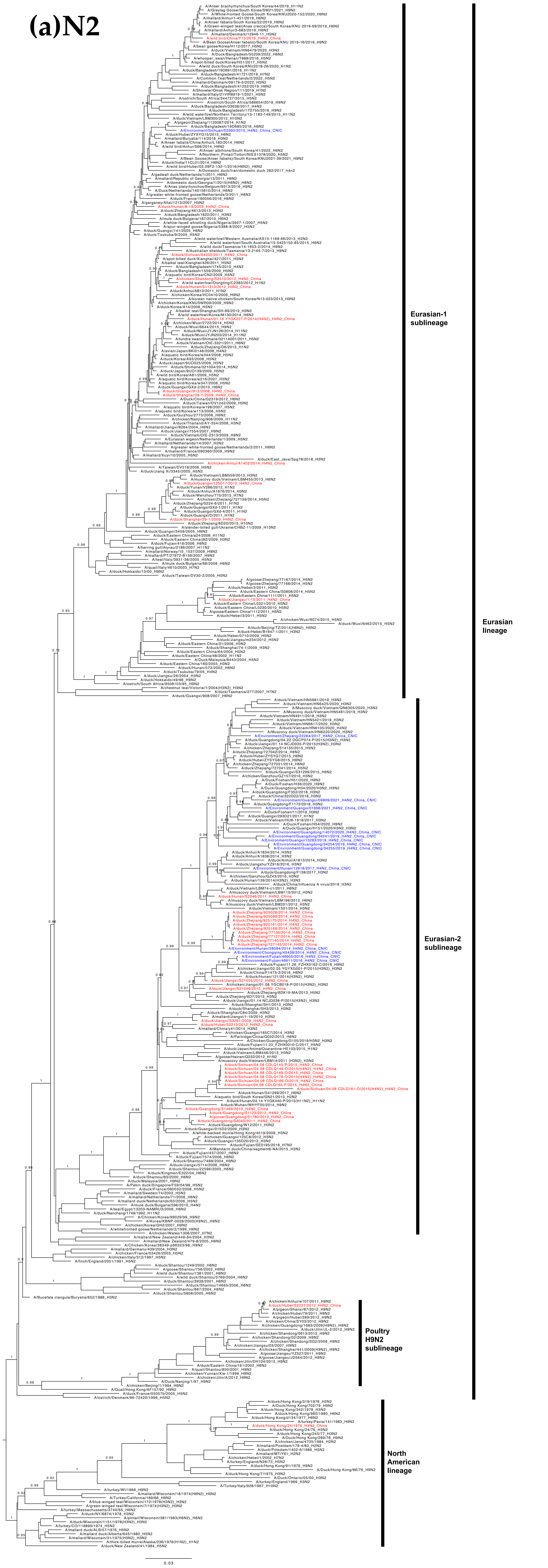
(a)N2
Eurasian-1 sublineage
Eurasian lineage
Eurasian-2 sublineage
Poultry H9N2 sublineage
North American lineage
0.03

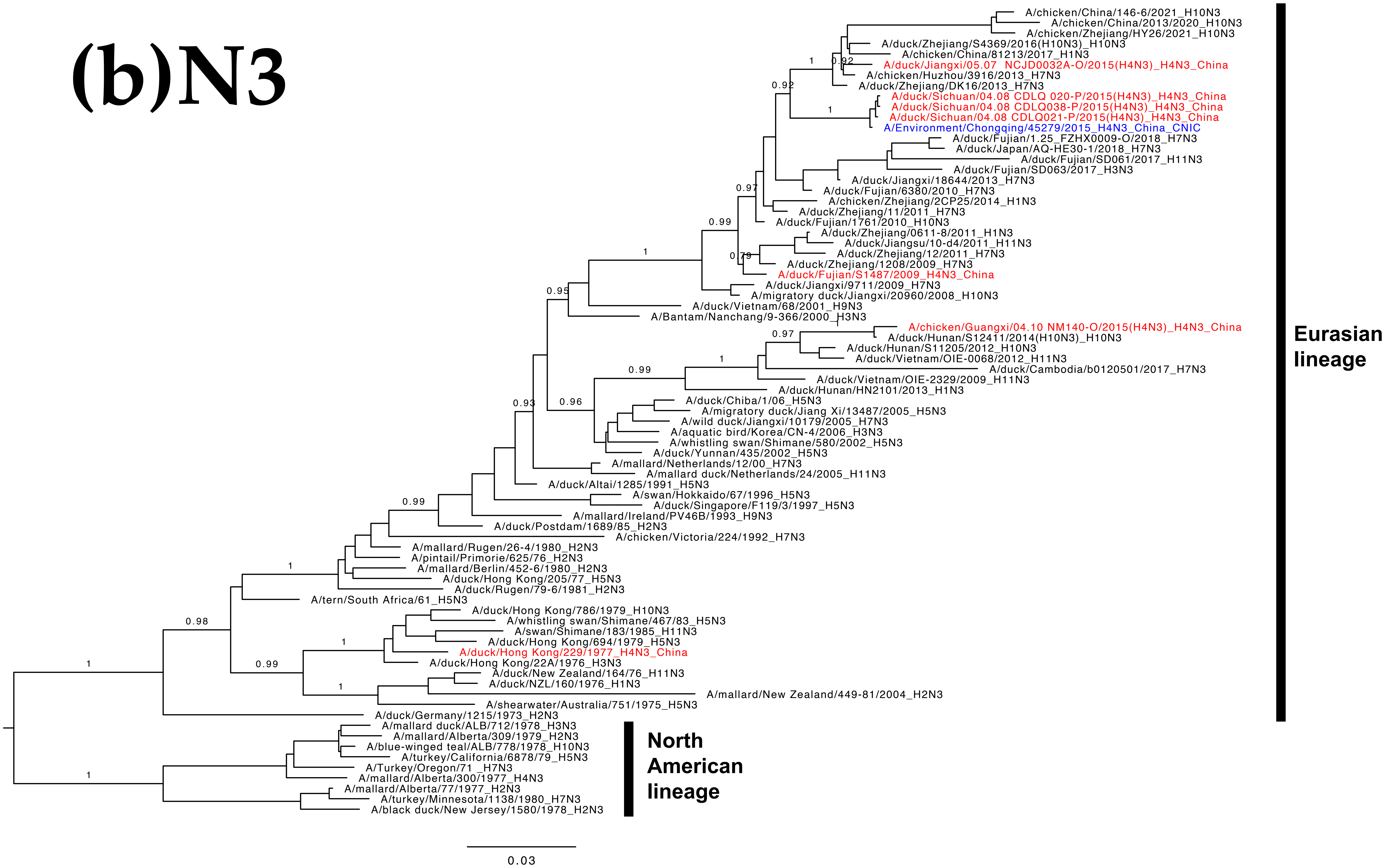

(b)N3
A/chicken/China/146-6/2021_H10N3
A/chicken/China/2013/2020_H10N3
A/chicken/Zhejiang/HY26/2021_H10N3
A/duck/Zhejiang/S4369/2016(H10N3)_H10N3
A/chicken/China/81213/2017_H1N3
A/duck/Jiangxi/05.07_NCJD0032A-O/2015(H4N3)_H4N3_China
A/chicken/Huzhou/3916/2013_H7N3
A/duck/Zhejiang/DK16/2013_H7N3
A/duck/Sichuan/04.08_CDLQ 020-P/2015(H4N3)_H4N3_China
A/duck/Sichuan/04.08_CDLQ038-P/2015(H4N3)_H4N3_China
A/duck/Sichuan/04.08_CDLQ021-P/2015(H4N3)_H4N3_China
A/Environment/Chongqing/45279/2015_H4N3_China_CNIC
A/duck/Fujian/1.25_FZHX0009-O/2018_H7N3
A/duck/Japan/AQ-HE30-1/2018_H7N3
A/duck/Fujian/SD061/2017_H11N3
A/duck/Fujian/SD063/2017_H3N3
A/duck/Jiangxi/18644/2013_H7N3
A/duck/Fujian/6380/2010_H7N3
A/chicken/Zhejiang/2CP25/2014_H1N3
A/duck/Zhejiang/11/2011_H7N3
A/duck/Fujian/1761/2010_H10N3
A/duck/Zhejiang/0611-8/2011_H1N3
A/duck/Jiangsu/10-d4/2011_H11N3
A/duck/Zhejiang/12/2011_H7N3
A/duck/Zhejiang/1208/2009_H7N3
A/duck/Fujian/S1487/2009_H4N3_China
A/duck/Jiangxi/9711/2009_H7N3
A/migratory duck/Jiangxi/20960/2008_H10N3
A/duck/Vietnam/68/2001_H9N3
A/Bantam/Nanchang/9-366/2000_H3N3
A/chicken/Guangxi/04.10_NM140-O/2015(H4N3)_H4N3_China
A/duck/Hunan/S12411/2014(H10N3)_H10N3
A/duck/Hunan/S11205/2012_H10N3
A/duck/Vietnam/OIE-0068/2012_H11N3
A/duck/Cambodia/b0120501/2017_H7N3
A/duck/Vietnam/OIE-2329/2009_H11N3
A/duck/Hunan/HN2101/2013_H1N3
A/duck/Chiba/1/06_H5N3
A/migratory duck/Jiang Xi/13487/2005_H5N3
A/wild duck/Jiangxi/10179/2005_H7N3
A/aquatic bird/Korea/CN-4/2006_H3N3
A/whistling swan/Shimane/580/2002_H5N3
A/duck/Yunnan/435/2002_H5N3
A/mallard/Netherlands/12/00_H7N3
A/mallard duck/Netherlands/24/2005_H11N3
A/duck/Altai/1285/1991_H5N3
A/swan/Hokkaido/67/1996_H5N3
A/duck/Singapore/F119/3/1997_H5N3
A/mallard/Ireland/PV46B/1993_H9N3
A/duck/Postdam/1689/85_H2N3
A/chicken/Victoria/224/1992_H7N3
A/mallard/Rugen/26-4/1980_H2N3
A/pintail/Primorie/625/76_H2N3
A/mallard/Berlin/452-6/1980_H2N3
A/duck/Hong Kong/205/77_H5N3
A/duck/Rugen/79-6/1981_H2N3
A/tern/South Africa/61_H5N3
A/duck/Hong Kong/786/1979_H10N3
A/whistling swan/Shimane/467/83_H5N3
A/swan/Shimane/183/1985_H11N3
A/duck/Hong Kong/694/1979_H5N3
A/duck/Hong Kong/229/1977_H4N3_China
A/duck/Hong Kong/22A/1976_H3N3
A/duck/New Zealand/164/76_H11N3
A/duck/NZL/160/1976_H1N3
A/mallard/New Zealand/449-81/2004_H2N3
A/shearwater/Australia/751/1975_H5N3
A/duck/Germany/1215/1973_H2N3
A/mallard duck/ALB/712/1978_H3N3
A/mallard/Alberta/309/1979_H2N3
A/blue-winged teal/ALB/778/1978_H10N3
A/turkey/California/6878/79_H5N3
A/Turkey/Oregon/71 _H7N3
A/mallard/Alberta/300/1977_H4N3
A/mallard/Alberta/77/1977_H2N3
A/turkey/Minnesota/1138/1980_H7N3
A/black duck/New Jersey/1580/1978_H2N3
Eurasian lineage
North American lineage
0.03

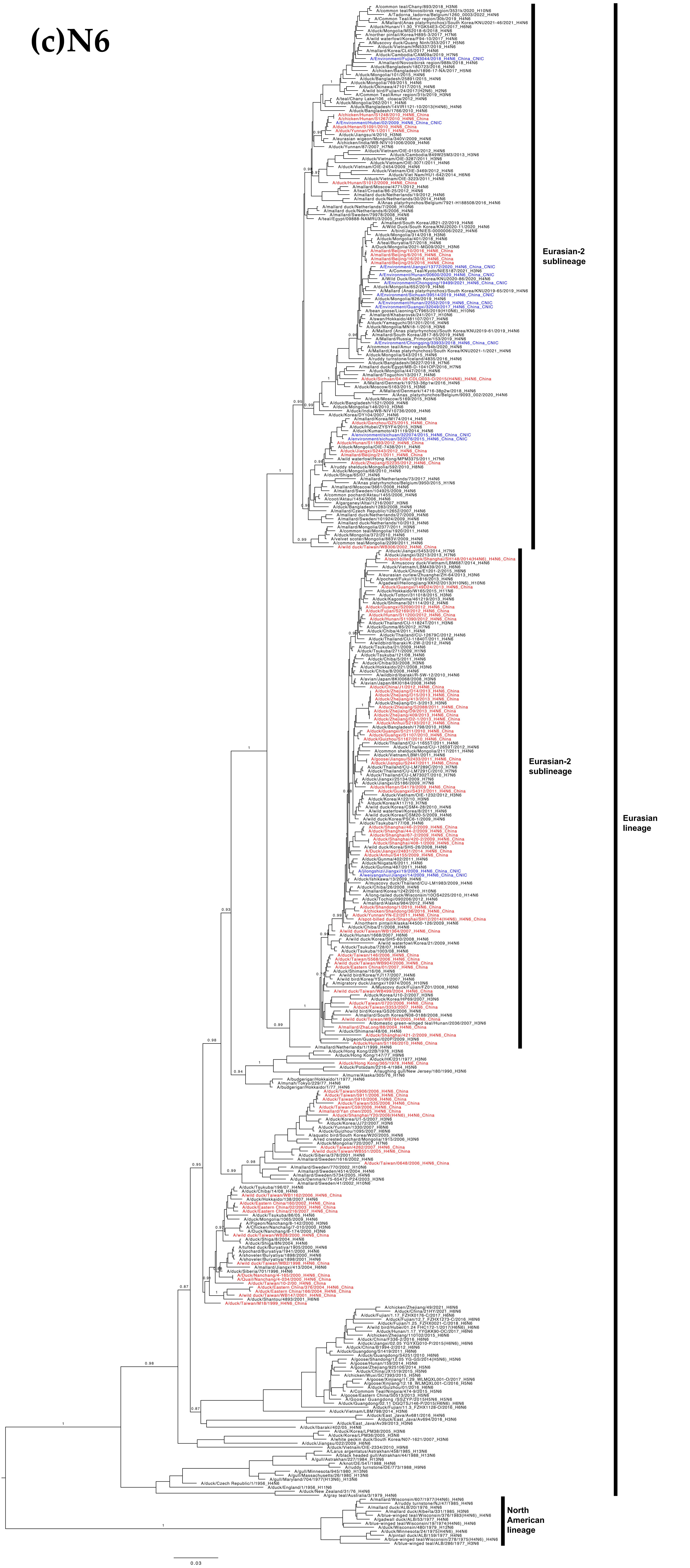
(c)N6
Eurasian-2 sublineage
Eurasian-2 sublineage
Eurasian lineage
North American lineage
0.03

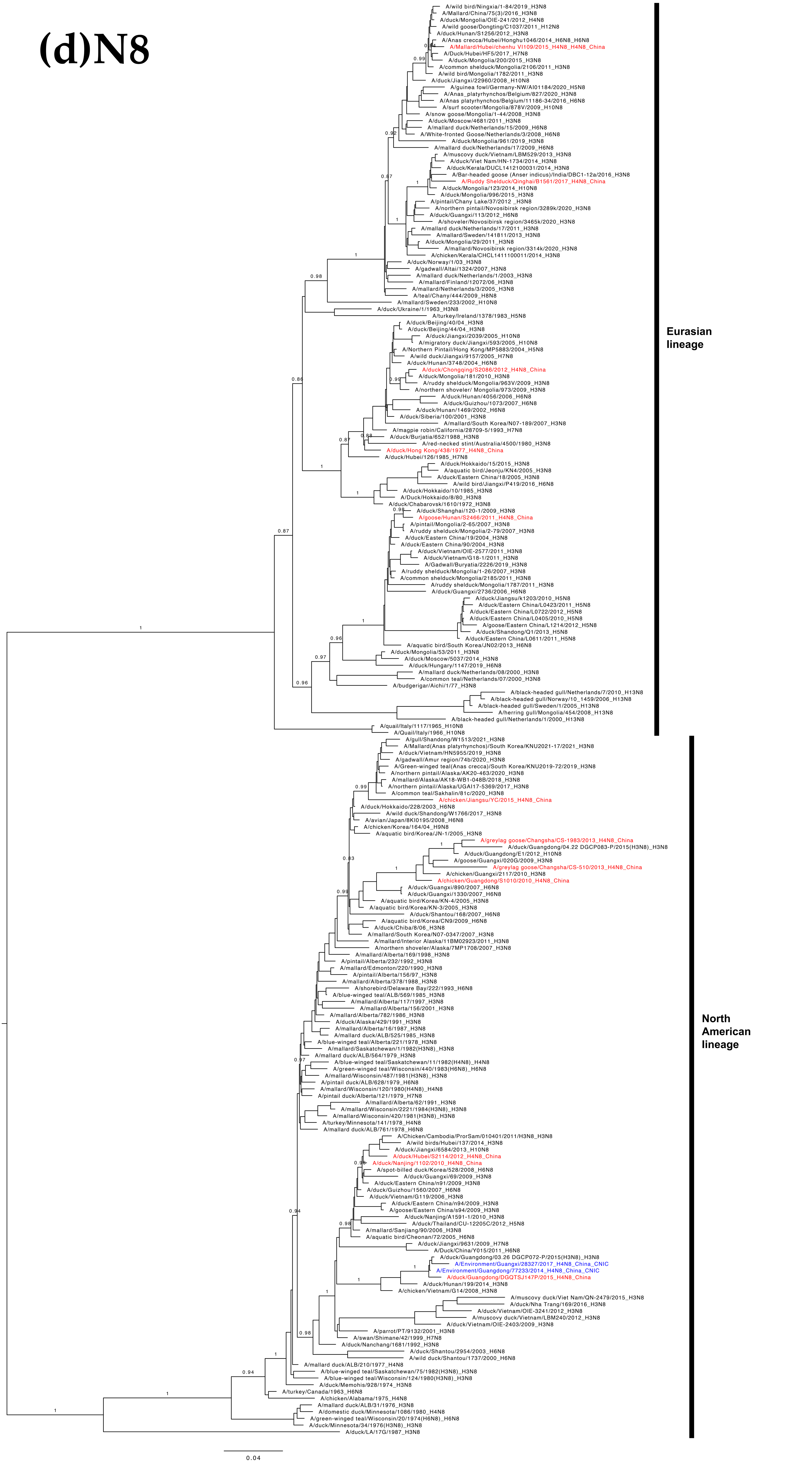
(d)N8
A/wild bird/Ningxia/1-84/2019_H3N8
A/Mallard/China/75(3)/2016_H3N8
A/duck/Mongolia/OIE-241/2012_H4N8
A/wild goose/Dongting/C1037/2011_H12N8
A/duck/Hunan/S1256/2012_H3N8
A/Anas crecca/Hubei/Honghu1046/2014_H6N8_H6N8
A/Mallard/Hubei/chenhu VI109/2015_H4N8_H4N8_China
A/Duck/Hubei/HF5/2017_H7N8
A/duck/Mongolia/200/2015_H3N8
A/common shelduck/Mongolia/2106/2011_H3N8
A/wild bird/Mongolia/1782/2011_H3N8
A/duck/Jiangxi/22960/2008_H10N8
A/guinea fowl/Germany-NW/AI01184/2020_H5N8
A/Anas_platyrhynchos/Belgium/827/2020_H3N8
A/Anas platyrhynchos/Belgium/11186-34/2016_H6N8
A/surf scooter/Mongolia/878V/2009_H10N8
A/snow goose/Mongolia/1-44/2008_H3N8
A/duck/Moscow/4681/2011_H3N8
A/mallard duck/Netherlands/15/2009_H6N8
A/White-fronted Goose/Netherlands/3/2008_H6N8
A/duck/Mongolia/961/2019_H3N8
A/mallard duck/Netherlands/17/2009_H6N8
A/muscovy duck/Vietnam/LBM529/2013_H3N8
A/duck/Viet Nam/HN-1734/2014_H3N8
A/duck/Kerala/DUCL1412100031/2014_H3N8
A/Bar-headed goose (Anser indicus)/India/DBC1-12a/2016_H3N8
A/Ruddy Shelduck/Qinghai/B1561/2017_H4N8_China
A/duck/Mongolia/123/2014_H10N8
A/duck/Mongolia/996/2015_H3N8
A/pintail/Chany Lake/37/2012_H3N8
A/northern pintail/Novosibirsk region/3289k/2020_H3N8
A/duck/Guangxi/113/2012_H6N8
A/shoveler/Novosibirsk region/3465k/2020_H3N8
A/mallard duck/Netherlands/17/2011_H3N8
A/mallard/Sweden/141811/2013_H3N8
A/duck/Mongolia/29/2011_H3N8
A/mallard/Novosibirsk region/3314k/2020_H3N8
A/chicken/Kerala/CHCL1411100011/2014_H3N8
A/duck/Norway/1/03_H3N8
A/gadwall/Altai/1324/2007_H3N8
A/mallard duck/Netherlands/1/2003_H3N8
A/mallard/Finland/12072/06_H3N8
A/mallard/Netherlands/3/2005_H3N8
A/teal/Chany/444/2009_H8N8
A/mallard/Sweden/233/2002_H10N8
A/duck/Ukraine/1/1963_H3N8
A/turkey/Ireland/1378/1983_H5N8
A/duck/Beijing/40/04_H3N8
A/duck/Beijing/44/04_H3N8
A/duck/Jiangxi/2039/2005_H10N8
A/migratory duck/Jiangxi/593/2005_H10N8
A/Northern Pintail/Hong Kong/MP5883/2004_H5N8
A/wild duck/Jiangxi/9157/2005_H7N8
A/duck/Hunan/3748/2004_H6N8
A/duck/Chongqing/S2086/2012_H4N8_China
A/duck/Mongolia/181/2010_H3N8
A/ruddy shelduck/Mongolia/963V/2009_H3N8
A/northern shoveler/ Mongolia/973/2009_H3N8
A/duck/Hunan/4056/2006_H6N8
A/duck/Guizhou/1073/2007_H6N8
A/duck/Hunan/1469/2002_H6N8
A/duck/Siberia/100/2001_H3N8
A/mallard/South Korea/N07-189/2007_H3N8
A/magpie robin/California/28709-5/1993_H7N8
A/duck/Burjatia/652/1988_H3N8
A/red-necked stint/Australia/4500/1980_H3N8
A/duck/Hong Kong/438/1977_H4N8_China
A/duck/Hubei/126/1985_H7N8
A/duck/Hokkaido/15/2015_H3N8
A/aquatic bird/Jeonju/KN4/2005_H3N8
A/duck/Eastern China/18/2005_H3N8
A/wild bird/Jiangxi/P419/2016_H6N8
A/duck/Hokkaido/10/1985_H3N8
A/Duck/Hokkaido/8/80_H3N8
A/duck/Chabarovsk/1610/1972_H3N8
A/duck/Shanghai/120-1/2009_H3N8
A/goose/Hunan/S2466/2011_H4N8_China
A/pintail/Mongolia/2-65/2007_H3N8
A/ruddy shelduck/Mongolia/2-79/2007_H3N8
A/duck/Eastern China/19/2004_H3N8
A/duck/Eastern China/90/2004_H3N8
A/duck/Vietnam/OIE-2577/2011_H3N8
A/duck/Vietnam/G18-1/2011_H3N8
A/Gadwall/Buryatia/2226/2019_H3N8
A/ruddy shelduck/Mongolia/1-26/2007_H3N8
A/common shelduck/Mongolia/2185/2011_H3N8
A/ruddy shelduck/Mongolia/1787/2011_H3N8
A/duck/Guangxi/2736/2006_H6N8
A/duck/Jiangsu/k1203/2010_H5N8
A/duck/Eastern China/L0423/2011_H5N8
A/duck/Eastern China/L0722/2012_H5N8
A/duck/Eastern China/L0405/2010_H5N8
A/goose/Eastern China/L1214/2012_H5N8
A/duck/Shandong/Q1/2013_H5N8
A/duck/Eastern China/L0611/2011_H5N8
A/aquatic bird/South Korea/JN02/2013_H6N8
A/duck/Mongolia/53/2011_H3N8
A/duck/Moscow/5037/2014_H3N8
A/duck/Hungary/1147/2019_H6N8
A/mallard duck/Netherlands/08/2000_H3N8
A/common teal/Netherlands/07/2000_H3N8
A/budgerigar/Aichi/1/77_H3N8
A/black-headed gull/Netherlands/7/2010_H13N8
A/black-headed gull/Norway/10_1459/2006_H13N8
A/black-headed gull/Sweden/1/2005_H13N8
A/herring gull/Mongolia/454/2008_H13N8
A/black-headed gull/Netherlands/1/2000_H13N8
A/quail/Italy/1117/1965_H10N8
A/Quail/Italy/1966_H10N8
Eurasian lineage
A/gull/Shandong/W1513/2021_H3N8
A/Mallard(Anas platyrhynchos)/South Korea/KNU2021-17/2021_H3N8
A/duck/Vietnam/HN5955/2019_H3N8
A/gadwall/Amur region/74b/2020_H3N8
A/Green-winged teal(Anas crecca)/South Korea/KNU2019-72/2019_H3N8
A/northern pintail/Alaska/AK20-463/2020_H3N8
A/mallard/Alaska/AK18-WB1-048B/2018_H3N8
A/northern pintail/Alaska/UGAI17-5369/2017_H3N8
A/common teal/Sakhalin/81c/2020_H3N8
A/chicken/Jiangsu/YC/2015_H4N8_China
A/duck/Hokkaido/228/2003_H6N8
A/wild duck/Shandong/W1766/2017_H3N8
A/avian/Japan/8KI0195/2008_H6N8
A/chicken/Korea/164/04_H9N8
A/aquatic bird/Korea/JN-1/2005_H3N8
A/greylag goose/Changsha/CS-1983/2013_H4N8_China
A/duck/Guangdong/04.22 DGCP083-P/2015(H3N8)_H3N8
A/duck/Guangdong/E1/2012_H10N8
A/goose/Guangxi/020G/2009_H3N8
A/greylag goose/Changsha/CS-510/2013_H4N8_China
A/chicken/Guangxi/2117/2010_H3N8
A/chicken/Guangdong/S1010/2010_H4N8_China
A/duck/Guangxi/890/2007_H6N8
A/duck/Guangxi/1330/2007_H6N8
A/aquatic bird/Korea/KN-4/2005_H3N8
A/aquatic bird/Korea/KN-3/2005_H3N8
A/duck/Shantou/168/2007_H6N8
A/aquatic bird/Korea/CN9/2009_H6N8
A/duck/Chiba/8/06_H3N8
A/mallard/South Korea/N07-0347/2007_H3N8
A/mallard/Interior Alaska/11BM02923/2011_H3N8
A/northern shoveler/Alaska/7MP1708/2007_H3N8
A/mallard/Alberta/169/1998_H3N8
A/pintail/Alberta/232/1992_H3N8
A/mallard/Edmonton/220/1990_H3N8
A/pintail/Alberta/156/97_H3N8
A/mallard/Alberta/378/1988_H3N8
A/shorebird/Delaware Bay/222/1993_H6N8
A/blue-winged teal/ALB/569/1985_H3N8
A/mallard/Alberta/117/1997_H3N8
A/mallard/Alberta/156/2001_H3N8
A/mallard/Alberta/782/1986_H3N8
A/duck/Alaska/429/1991_H3N8
A/mallard/Alberta/16/1987_H3N8
A/mallard duck/ALB/525/1985_H3N8
A/blue-winged teal/Alberta/221/1978_H3N8
A/mallard/Saskatchewan/1/1982(H3N8)_H3N8
A/mallard duck/ALB/564/1979_H3N8
A/blue-winged teal/Saskatchewan/11/1982(H4N8)_H4N8
A/green-winged teal/Wisconsin/440/1983(H6N8)_H6N8
A/mallard/Wisconsin/487/1981(H3N8)_H3N8
A/pintail duck/ALB/628/1979_H6N8
A/mallard/Wisconsin/120/1980(H4N8)_H4N8
A/pintail duck/Alberta/121/1979_H7N8
A/mallard/Alberta/62/1991_H3N8
A/mallard/Wisconsin/2221/1984(H3N8)_H3N8
A/mallard/Wisconsin/420/1981(H3N8)_H3N8
A/turkey/Minnesota/141/1978_H4N8
A/mallard duck/ALB/761/1978_H6N8
A/Chicken/Cambodia/ProrSam/010401/2011/H3N8_H3N8
A/wild birds/Hubei/137/2014_H3N8
A/duck/Jiangxi/6584/2013_H10N8
A/duck/Hubei/S2114/2012_H4N8_China
A/duck/Nanjing/1102/2010_H4N8_China
A/spot-billed duck/Korea/528/2008_H6N8
A/duck/Guangxi/69/2009_H3N8
A/duck/Eastern China/n91/2009_H3N8
A/duck/Guizhou/1560/2007_H6N8
A/duck/Vietnam/G119/2006_H3N8
A/duck/Eastern China/n94/2009_H3N8
A/goose/Eastern China/s94/2009_H3N8
A/duck/Nanjing/A1591-1/2010_H3N8
A/duck/Thailand/CU-12205C/2012_H5N8
A/mallard/Sanjiang/90/2006_H3N8
A/aquatic bird/Cheonan/72/2005_H6N8
A/duck/Jiangxi/9631/2009_H7N8
A/Duck/China/Y015/2011_H6N8
A/duck/Guangdong/03.26 DGCP072-P/2015(H3N8)_H3N8
A/Environment/Guangxi/28327/2017_H4N8_China_CNIC
A/Environment/Guangdong/77233/2014_H4N8_China_CNIC
A/duck/Guangdong/DGQTSJ147P/2015_H4N8_China
A/duck/Hunan/199/2014_H3N8
A/chicken/Vietnam/G14/2008_H3N8
A/muscovy duck/Viet Nam/QN-2479/2015_H3N8
A/duck/Nha Trang/169/2016_H3N8
A/duck/Vietnam/OIE-3241/2012_H3N8
A/muscovy duck/Vietnam/LBM240/2012_H3N8
A/duck/Vietnam/OIE-2403/2009_H3N8
A/parrot/PT/9132/2001_H3N8
A/swan/Shimane/42/1999_H7N8
A/duck/Nanchang/1681/1992_H3N8
A/duck/Shantou/2954/2003_H6N8
A/wild duck/Shantou/1737/2000_H6N8
A/mallard duck/ALB/210/1977_H4N8
A/blue-winged teal/Saskatchewan/75/1982(H3N8)_H3N8
A/blue-winged teal/Wisconsin/124/1980(H3N8)_H3N8
A/duck/Memphis/928/1974_H3N8
A/turkey/Canada/1963_H6N8
A/chicken/Alabama/1975_H4N8
A/mallard duck/ALB/31/1976_H3N8
A/domestic duck/Minnesota/1086/1980_H4N8
A/green-winged teal/Wisconsin/20/1974(H6N8)_H6N8
A/duck/Minnesota/34/1976(H3N8)_H3N8
A/duck/LA/17G/1987_H3N8
North American lineage
0.04

Figure S2. Phylogenetic tree of neuraminidase (NA) genes. (a) N2 gene (n=385); (b) N3 gene (n=77); (c) N6 gene (n=386); (d) N8 gene (n=213). H4 strains sequenced in this study are in steel blue. Other H4 AIVs in China are in red. Branch lengths are scaled according to the number of substitutions per site. Branch support values of selected nodes are shown. Lineages, sublineages and subgroups are labeled with vertical lines on the right.
