# Supplementary material for: Cross-Species Transmission Potential of H4 Avian Influenza Viruses in China: Epidemiological and Evolutionary Study
Source: Viruses. 2024 Feb 24;16(3):353. doi: 10.3390/v16030353 (PMC10974465; doi:10.3390/v16030353)
Supplement: Supplementary file 1 [file viruses-16-00353-s001.zip › Supplementary Figure S3.pdf]

(a)PB2

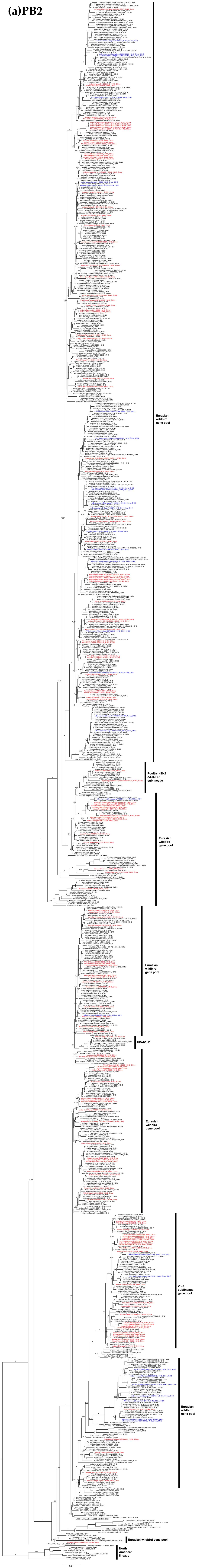
Eurasian wildbird gene pool
Poultry H9N2 ZJ-HJ/07 sublineage
Eurasian wildbird gene pool
Eurasian wildbird gene pool
HPAIV H5
Eurasian wildbird gene pool
ZJ-5 sublineage gene pool
Eurasian wildbird gene pool
Eurasian wildbird gene pool
North American lineage

(b)PB1

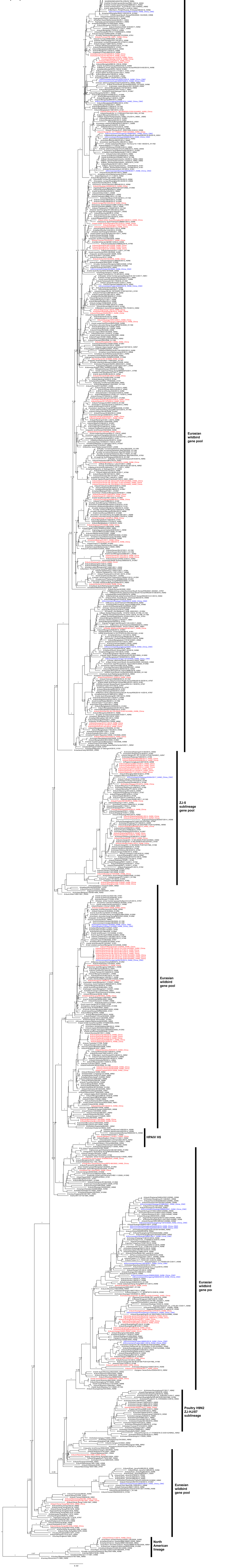

Eurasian wildbird gene pool
ZJ-5 sublineage gene pool
Eurasian wildbird gene pool
HPAIV H5
Eurasian wildbird gene pool
Poultry H9N2 ZJ-HJ/07 sublineage
Eurasian wildbird gene pool
North American lineage

(c)PA

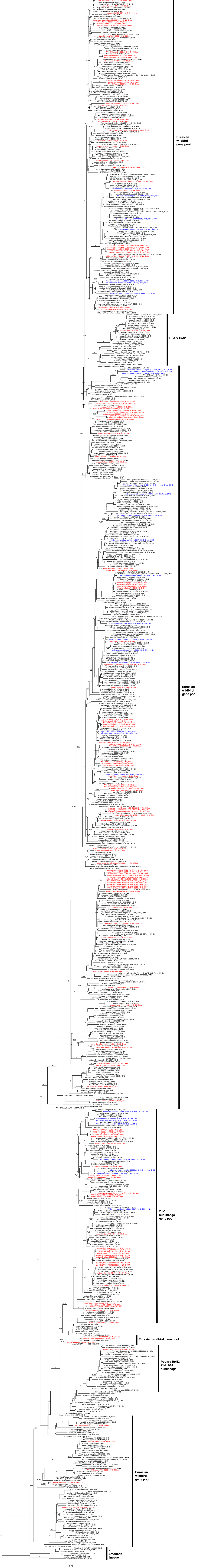

Eurasian wildbird gene pool
HPAIV H5N1
Eurasian wildbird gene pool
ZJ-5 sublineage gene pool
Eurasian wildbird gene pool
Poultry H9N2 ZJ-HJ/07 sublineage
Eurasian wildbird gene pool
North American lineage

(d)NP

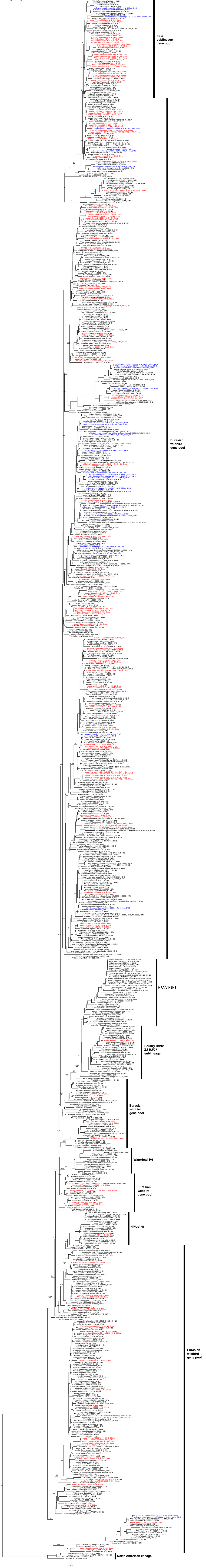
ZJ-5 sublineage gene pool
Eurasian wildbird gene pool
HPAIV H5N1
Poultry H9N2 ZJ-HJ07 sublineage
Eurasian wildbird gene pool
Waterfowl H6
Eurasian wildbird gene pool
HPAIV H5
Eurasian wildbird gene pool
North American lineage

(e)M

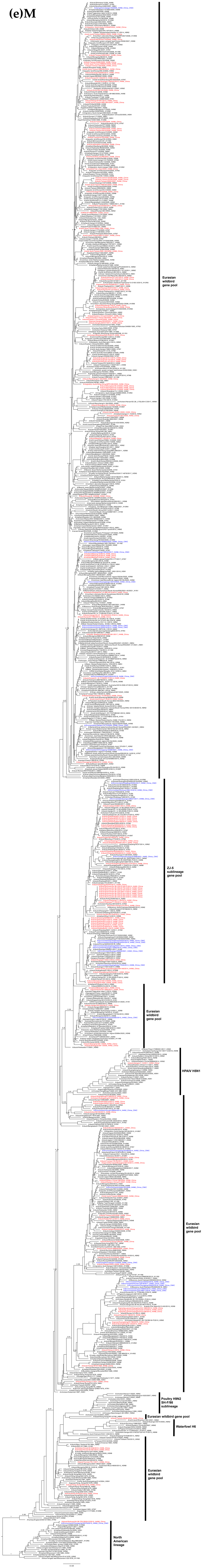
Eurasian wildbird gene pool
ZJ-5 sublineage gene pool
Eurasian wildbird gene pool
HPAIV H5N1
Eurasian wildbird gene pool
Poultry H9N2 SH-F/98 sublineage
Eurasian wildbird gene pool
Waterfowl H6
Eurasian wildbird gene pool
North American lineage

(f)NS

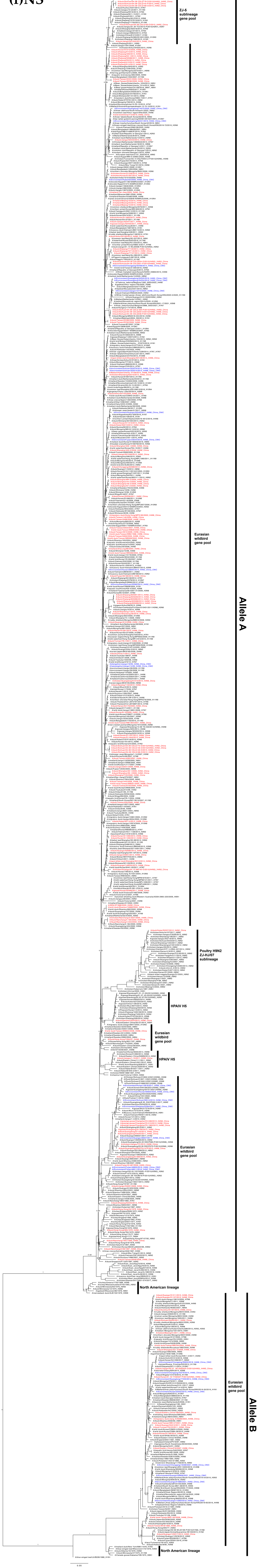
ZJ-5 sublineage gene pool
Eurasian wildbird gene pool
Allele A
Poultry H9N2 ZJ-HJ/07 sublineage
HPAIV H5
Eurasian wildbird gene pool
HPAIV H5
Eurasian wildbird gene pool
North American lineage
Eurasian wildbird gene pool
Allele B
North American lineage

Figure S3. Maximum likelihood trees of internal-protein coding genes. (a) PB2 gene (n=937); (b) PB1 gene (n=929); (c) PA gene (n=890); (d) NP gene (n=914); (e) M gene (n=890); (f) NS gene (n=749). H4 strains sequenced in this study are in steel blue. Other H4 AIVs in China are in red. Branch lengths are scaled according to the number of substitutions per site. Branch support values of selected nodes are shown. Lineages, sublineages and subgroups are labeled with vertical lines on the right.
