# Supplementary material for: Cross-Species Transmission Potential of H4 Avian Influenza Viruses in China: Epidemiological and Evolutionary Study
Source: Viruses. 2024 Feb 24;16(3):353. doi: 10.3390/v16030353 (PMC10974465; doi:10.3390/v16030353)
Supplement: Supplementary file 1 [file viruses-16-00353-s001.zip › Supplementary Figure S4.pdf]

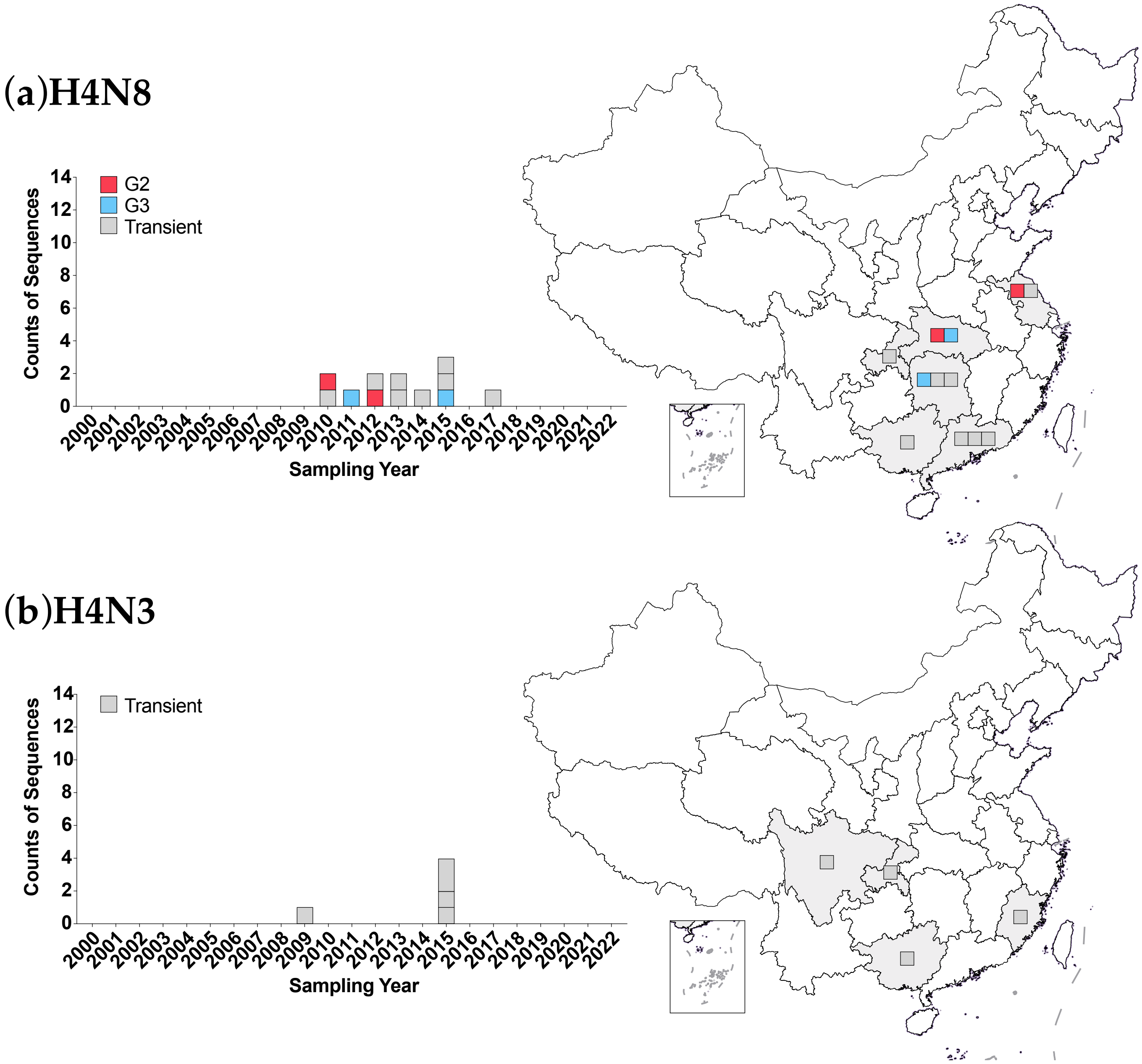

Figure 4. Diversity of genotypes of H4N8 and H4N3 AIVs in China during 2000-2022. (a) The distribution of genotypes of H4N8; (b) The distribution of genotypes of H4N3. The left panel shows the temporal distribution of the genotypes. The right panel shows the geographic distribu-tion of the genotypes; provinces with detected genotypes are filled with grey color on the map; different genotypes are marked with square symbols of corresponding color on the map; transient genotypes are marked with grey square symbols.
