# Supplementary material for: Cross-Species Transmission Potential of H4 Avian Influenza Viruses in China: Epidemiological and Evolutionary Study
Source: Viruses. 2024 Feb 24;16(3):353. doi: 10.3390/v16030353 (PMC10974465; doi:10.3390/v16030353)
Supplement: Supplementary file 1 [file viruses-16-00353-s001.zip › Supplementary Table 1.pdf]

Table S1. Detailed information of 31 environmental H4 subtype viruses obtained in our surveillance.

Subtype	Isolate name	Province	Collection date	Sampling site*	Sampling type
H4N2	A/Environment/Hunan/28094/2014	Hunan	2014-04-21	live poultry market	others
	A/Environment/Chongqing/45438/2014	Chongqing	2014-12-17	live poultry market	others
	A/Environment/Sichuan/03392/2015	Sichuan	2015-12-09	live poultry market	others
	A/Environment/Fujian/46605/2016	Fujian	2016-02-22	live poultry market	others
	A/Environment/Fujian/46611/2016	Fujian	2016-02-22	live poultry market	others
	A/Environment/Hunan/12618/2017	Hunan	2017-02-08	live poultry market	others
	A/Environment/Zhejiang/22264/2017	Zhejiang	2017-12-22	poultry processing companies	others
	A/Environment/Guangxi/13283/2019	Guangxi	2019-01-17	live poultry market	others
	A/Environment/Guangdong/34241/2019	Guangdong	2019-02-13	live poultry market	feces
	A/Environment/Guangdong/34255/2019	Guangdong	2019-03-25	live poultry market	feces
	A/Environment/Guangdong/34254/2019	Guangdong	2019-03-25	live poultry market	drinking water
	A/Environment/Guangdong/14072/2020	Guangdong	2020-10-19	unknown	others
	A/Environment/Guangxi/01898/2021	Guangxi	2021-01-24	unknown	others
	A/Environment/Guangxi/09909/2021	Guangxi	2021-08-12	unknown	others
H4N3	A/Environment/Chongqing/45279/2015	Chongqing	2015-05-20	live poultry market	others
H4N6	A/jilongshizi/Jiangxi/19/2009	Jiangxi	2009-02-26	live poultry market	surface swab of cages
	A/weiyangshui/Jiangxi/14/2009	Jiangxi	2009-02-26	live poultry market	drinking water
	A/Environment/Hubei/02/2009	Hubei	2009-12-02	unknown	feces
	A/environment/sichuan/322074/2015	Sichuan	2015-07-24	poultry farm	sewage
	A/environment/sichuan/322076/2015	Sichuan	2015-07-24	poultry farm	sewage
	A/Environment/Guangxi/32049/2017	Guangxi	2017-05-25	live poultry market	others
	A/Environment/Fujian/23044/2018	Fujian	2018-01-08	live poultry market	others
	A/Environment/Chongqing/33933/2018	Chongqing	2018-09-17	live poultry market	surface swab of cages
	A/Environment/Hunan/22552/2019	Hunan	2019-01-08	live poultry market	drinking water
	A/Environment/Sichuan/39514/2019	Sichuan	2019-08-02	unknown	others
	A/Environment/Jiangxi/13772/2020	Jiangxi	2020-12-08	unknown	others

	A/Environment/Hunan/00600/2020	Hunan	2020-12-24	unknown	others
	A/Environment/Chongqing/19499/2021	Chongqing	2021-11-24	live poultry market	others
H4N8	A/Environment/Guangdong/77233/2014	Guangdong	2014-08-18	live poultry market	others
	A/Environment/Guangxi/28327/2017	Guangxi	2017-05-04	live poultry market	others
H4	A/Environment/Jiangxi/33289/2015(mixed)	Jiangxi	2015-03-24	live poultry market	others

* Unknown, referred to sampling sites without specific information (live poultry market, poultry farm, backyard or processing plants).
