# Supplementary material for: Cross-Species Transmission Potential of H4 Avian Influenza Viruses in China: Epidemiological and Evolutionary Study
Source: Viruses. 2024 Feb 24;16(3):353. doi: 10.3390/v16030353 (PMC10974465; doi:10.3390/v16030353)
Supplement: Supplementary file 1 [file viruses-16-00353-s001.zip › Supplementary Table 2.pdf]

Table S2. Genotypes of all H4 subtype AIVs with the whole genome*.

Subtype	Isolate name	Host	Genotype	HA	NA	PB2	PB1	PA	NP	M	NS
**H4N2**	A/duck/Hong Kong/24/1976	wild birds	G1	Eurasian-1 sublineage	N2-North American lineage	EA	EA	EA	EA	EA	EA(A)
	A/duck/Guangxi/912/2008	poultry	G2	Eurasian-1 sublineage	N2-Eurasian-1 sublineage	EA	EA	EA	EA	EA	EA(A)
	A/Environment/Sichuan/03392/2015	poultry-related environment	G2	Eurasian-1 sublineage	N2-Eurasian-1 sublineage	EA	EA	EA	EA	EA	EA(A)
	A/duck/Jiangxi/S3261/2009	poultry	G3	Eurasian-1 sublineage	N2-Eurasian-2 sublineage	EA	EA	EA	EA	EA	EA(A)
	A/duck/Guangdong/S1469/2010	poultry	G3	Eurasian-1 sublineage	N2-Eurasian-2 sublineage	EA	EA	EA	EA	EA	EA(A)
	A/duck/Guangdong/S1123/2012	poultry	G3	Eurasian-1 sublineage	N2-Eurasian-2 sublineage	EA	EA	EA	EA	EA	EA(A)
	A/goose/Guangdong/S1780/2012	poultry	G3	Eurasian-1 sublineage	N2-Eurasian-2 sublineage	EA	EA	EA	EA	EA	EA(A)
	A/duck/Hunan/8-19/2009	poultry	G4	Eurasian-2 sublineage	N2-Eurasian-1 sublineage	EA	EA	EA	EA	EA	EA(A)
	A/duck/Hunan/S11313/2012	poultry	G4	Eurasian-2 sublineage	N2-Eurasian-1 sublineage	EA	EA	EA	EA	EA	EA(A)
	A/chicken/Shandong/S2510/2012	poultry	G4	Eurasian-2 sublineage	N2-Eurasian-1 sublineage	EA	EA	EA	EA	EA	EA(A)
	A/wild bird/China/Y13/2019	wild birds	G4	Eurasian-2 sublineage	N2-Eurasian-1 sublineage	EA	EA	EA	EA	EA	EA(A)
	A/duck/Shanghai/29-1/2009	poultry	G5	Eurasian-2 sublineage	N2-Eurasian-1 sublineage	ZJ-5	EA	ZJ-5	ZJ-5	EA	EA(A)
	A/duck/Guangdong/S4040/2011	poultry	G6	Eurasian-1 sublineage	N2-Eurasian-2 sublineage	EA	EA	EA	ZJ-5	ZJ-5	EA(A)
	A/duck/Hunan/S2046/2011	poultry	G7	Eurasian-2 sublineage	N2-Eurasian-2 sublineage	EA	EA	ZJ-5	EA	EA	EA(A)
	A/Environment/Fujian/46611/2016	poultry-related environment	G7	Eurasian-2 sublineage	N2-Eurasian-2 sublineage	EA	EA	ZJ-5	EA	EA	EA(A)
	A/Environment/Fujian/46605/2016	poultry-related environment	G7	Eurasian-2 sublineage	N2-Eurasian-2 sublineage	EA	EA	ZJ-5	EA	EA	EA(A)
	A/duck/Sichuan/S4202/2011	poultry	G8	Eurasian-2 sublineage	N2-Eurasian-1 sublineage	EA	EA	EA	EA	EA	EA(B)
	A/duck/Jiangsu/1-15/2011	poultry	G9	Eurasian-2 sublineage	N2-Eurasian-1 sublineage	H5Nx	H5Nx	H5Nx	H5Nx	H5Nx	H5Nx
	A/duck/Hubei/S2213/2012	poultry	G10	Eurasian-2 sublineage	N2-Eurasian-2 sublineage	EA	EA	EA	EA	EA	EA(A)
	A/Environment/Chongqing/45438/2014	poultry-related environment	G10	Eurasian-2 sublineage	N2-Eurasian-2 sublineage	EA	EA	EA	EA	EA	EA(A)
	A/duck/Jiangxi/S21055/2012	poultry	G11	Eurasian-2 sublineage	N2-Eurasian-2 sublineage	EA	EA	ZJ-5	ZJ-5	EA	ZJ-5
	A/duck/Jiangxi/S21046/2012	poultry	G12	Eurasian-2 sublineage	N2-Eurasian-2 sublineage	ZJ-5	EA	ZJ-5	ZJ-5	ZJ-5	EA(A)

	A/duck/Guangxi/125D17/2012	poultry	G13	Eurasian-2 sublineage	N2-Eurasian-1 sublineage	ZJ-5	EA	EA	EA	ZJ-5	EA(B)
	A/duck/Hubei/S2227/2012	poultry	G14	Eurasian-2 sublineage	poultry H9N2 sublineage	H9N2	H9N2	H9N2	H9N2	EA	H9N2
	A/duck/Hunan/01.16 YYGK227-P/2014	poultry	G15	Eurasian-1 sublineage	N2-Eurasian-1 sublineage	EA	EA	EA	EA	EA	EA(B)
	A/Environment/Hunan/28094/2014	poultry-related environment	G16	Eurasian-2 sublineage	N2-Eurasian-2 sublineage	EA	EA	ZJ-5	ZJ-5	EA	EA(A)
	A/Environment/Hunan/12618/2017	poultry-related environment	G16	Eurasian-2 sublineage	N2-Eurasian-2 sublineage	EA	EA	ZJ-5	ZJ-5	EA	EA(A)
	A/duck/Zhejiang/925161/2014	poultry	G17	Eurasian-2 sublineage	N2-Eurasian-2 sublineage	EA	ZJ-5	ZJ-5	EA	ZJ-5	ZJ-5
	A/duck/Zhejiang/925170/2014	poultry	G17	Eurasian-2 sublineage	N2-Eurasian-2 sublineage	EA	ZJ-5	ZJ-5	EA	ZJ-5	ZJ-5
	A/duck/Zhejiang/77127/2014	poultry	G18	Eurasian-2 sublineage	N2-Eurasian-2 sublineage	ZJ-5	ZJ-5	ZJ-5	EA	ZJ-5	EA(A)
	A/duck/Zhejiang/77140/2014	poultry	G18	Eurasian-2 sublineage	N2-Eurasian-2 sublineage	ZJ-5	ZJ-5	ZJ-5	EA	ZJ-5	EA(A)
	A/duck/Zhejiang/727145/2014	poultry	G18	Eurasian-2 sublineage	N2-Eurasian-2 sublineage	ZJ-5	ZJ-5	ZJ-5	EA	ZJ-5	EA(A)
	A/Environment/Zhejiang/22264/2017	poultry-related environment	G18	Eurasian-2 sublineage	N2-Eurasian-2 sublineage	ZJ-5	ZJ-5	ZJ-5	EA	ZJ-5	EA(A)
	A/duck/Zhejiang/925028/2014	poultry	G19	Eurasian-2 sublineage	N2-Eurasian-2 sublineage	ZJ-5	ZJ-5	ZJ-5	ZJ-5	ZJ-5	EA(A)
	A/duck/Zhejiang/925088/2014	poultry	G19	Eurasian-2 sublineage	N2-Eurasian-2 sublineage	ZJ-5	ZJ-5	ZJ-5	ZJ-5	ZJ-5	EA(A)
	A/duck/Sichuan/04.08 CDLQ146-O/2015	poultry	G20	Eurasian-2 sublineage	N2-Eurasian-2 sublineage	EA	EA	EA	EA	ZJ-5	EA(A)
	A/Environment/Guangdong/34241/2019	poultry-related environment	G20	Eurasian-2 sublineage	N2-Eurasian-2 sublineage	EA	EA	EA	EA	ZJ-5	EA(A)
	A/Environment/Guangdong/34254/2019	poultry-related environment	G20	Eurasian-2 sublineage	N2-Eurasian-2 sublineage	EA	EA	EA	EA	ZJ-5	EA(A)
	A/duck/Sichuan/04.08 CDLQ169-O/2015	poultry	G21	Eurasian-2 sublineage	N2-Eurasian-2 sublineage	EA	EA	EA	EA	ZJ-5	ZJ-5
	A/duck/Sichuan/04.08 CDLQ145-P/2015	poultry	G21	Eurasian-2 sublineage	N2-Eurasian-2 sublineage	EA	EA	EA	EA	ZJ-5	ZJ-5
	A/Environment/Guangxi/13283/2019	poultry-related environment	G22	Eurasian-2 sublineage	N2-Eurasian-2 sublineage	EA	EA	EA	EA	ZJ-5	EA(B)
	A/Environment/Guangxi/01898/2021	poultry-related environment	G22	Eurasian-2 sublineage	N2-Eurasian-2 sublineage	EA	EA	EA	EA	ZJ-5	EA(B)
	A/Environment/Guangxi/09909/2021	poultry-related environment	G22	Eurasian-2 sublineage	N2-Eurasian-2 sublineage	EA	EA	EA	EA	ZJ-5	EA(B)
	A/Environment/Guangdong/34255/2019	poultry-related environment	G23	Eurasian-2 sublineage	N2-Eurasian-2 sublineage	EA	EA	ZJ-5	EA	ZJ-5	EA(A)
	A/Environment/Guangdong/14072/2020	poultry-related environment	G24	Eurasian-2 sublineage	N2-Eurasian-2 sublineage	EA	EA	EA	ZJ-5	EA	EA(A)
**H4N3**	A/duck/Fujian/S1487/2009	poultry	G1	Eurasian-1 sublineage	N3-Eurasian lineage	EA	EA	ZJ-5	EA	EA	EA(A)

	A/Environment/Chongqing/45279/2015	poultry-related environment	G2	Eurasian-1 sublineage	N3-Eurasian lineage	EA	EA	EA	EA	NA	EA(B)
	A/chicken/Guangxi/04.10 NM140-O/2015	poultry	G3	Eurasian-2 sublineage	N3-Eurasian lineage	EA	EA	ZJ-5	EA	EA	EA(A)
	A/duck/Sichuan/04.08 CDLQ 020-P/2015	poultry	G4	Eurasian-2 sublineage	N3-Eurasian lineage	EA	EA	EA	EA	ZJ-5	EA(A)
	A/duck/Sichuan/04.08 CDLQ021-P/2015	poultry	G4	Eurasian-2 sublineage	N3-Eurasian lineage	EA	EA	EA	EA	ZJ-5	EA(A)
**H4N6**	A/duck/Hong Kong/365/1978	wild birds	G1	Oceania−East Asian sublineage	N6-Eurasian lineage	EA	EA	EA	EA	EA	EA(A)
	A/Duck/Nanchang/4-165/2000	poultry	G2	Eurasian-1 sublineage	N6-Eurasian lineage	EA	EA	EA	EA	EA	EA(A)
	A/mallard/Yan chen/2005	wild birds	G2	Eurasian-1 sublineage	N6-Eurasian lineage	EA	EA	EA	EA	EA	EA(A)
	A/mallard/ZhaLong/88/2004	wild birds	G3	Eurasian-1 sublineage	N6-Eurasian-2 sublineage	EA	EA	EA	EA	EA	EA(A)
	A/duck/Shanghai/Y20/2006	poultry-related environment	G4	Eurasian-1 sublineage	N6-Eurasian lineage	EA	ZJ-5	EA	EA	EA	EA(A)
	A/duck/Hunan/S1012/2009	poultry	G5	Eurasian-1 sublineage	N6-Eurasian-1 sublineage	EA	EA	EA	EA	EA	EA(A)
	A/environment/sichuan/322076/2015	poultry-related environment	G5	Eurasian-1 sublineage	N6-Eurasian-1 sublineage	EA	EA	EA	EA	EA	EA(A)
	A/environment/sichuan/322074/2015	poultry-related environment	G5	Eurasian-1 sublineage	N6-Eurasian-1 sublineage	EA	EA	EA	EA	EA	EA(A)
	A/duck/Shanghai/421-2/2009	poultry	G6	Eurasian-1 sublineage	N6-Eurasian-2 sublineage	EA	EA	EA	EA	EA	EA(B)
	A/Environment/Hubei/02/2009	poultry-related environment	G7	Eurasian-2 sublineage	N6-Eurasian-1 sublineage	EA	EA	EA	EA	EA	EA(A)
	A/chicken/Hunan/S1248/2010	poultry	G7	Eurasian-2 sublineage	N6-Eurasian-1 sublineage	EA	EA	EA	EA	EA	EA(A)
	A/chicken/Hunan/S1267/2010	poultry	G7	Eurasian-2 sublineage	N6-Eurasian-1 sublineage	EA	EA	EA	EA	EA	EA(A)
	A/duck/Henan/S1091/2010	poultry	G7	Eurasian-2 sublineage	N6-Eurasian-1 sublineage	EA	EA	EA	EA	EA	EA(A)
	A/duck/Yunnan/YN-1/2011	wild birds	G7	Eurasian-2 sublineage	N6-Eurasian-1 sublineage	EA	EA	EA	EA	EA	EA(A)
	A/duck/Hunan/S11893/2012	poultry	G7	Eurasian-2 sublineage	N6-Eurasian-1 sublineage	EA	EA	EA	EA	EA	EA(A)
	A/duck/Jiangxi/S2443/2012	poultry	G7	Eurasian-2 sublineage	N6-Eurasian-1 sublineage	EA	EA	EA	EA	EA	EA(A)
	A/mallard/Beijing/10/2016	wild birds	G7	Eurasian-2 sublineage	N6-Eurasian-1 sublineage	EA	EA	EA	EA	EA	EA(A)
	A/mallard/Beijing/16/2016	wild birds	G7	Eurasian-2 sublineage	N6-Eurasian-1 sublineage	EA	EA	EA	EA	EA	EA(A)
	A/Environment/Guangxi/32049/2017	poultry-related environment	G7	Eurasian-2 sublineage	N6-Eurasian-1 sublineage	EA	EA	EA	EA	EA	EA(A)
	A/Environment/Fujian/23044/2018	poultry-related environment	G7	Eurasian-2 sublineage	N6-Eurasian-1 sublineage	EA	EA	EA	EA	EA	EA(A)

A/Environment/Jiangxi/13772/2020	poultry-related environment	G7	Eurasian-2 sublineage	N6-Eurasian-1 sublineage	EA	EA	EA	EA	EA	EA(A)
A/Environment/Hunan/00600/2020	poultry-related environment	G7	Eurasian-2 sublineage	N6-Eurasian-1 sublineage	EA	EA	EA	EA	EA	EA(A)
A/weiyangshui/Jiangxi/14/2009	poultry-related environment	G8	Eurasian-2 sublineage	N6-Eurasian-2 sublineage	EA	EA	EA	EA	EA	EA(A)
A/jilongshizi/Jiangxi/19/2009	poultry-related environment	G8	Eurasian-2 sublineage	N6-Eurasian-2 sublineage	EA	EA	EA	EA	EA	EA(A)
A/duck/Anhui/S4155/2009	poultry	G8	Eurasian-2 sublineage	N6-Eurasian-2 sublineage	EA	EA	EA	EA	EA	EA(A)
A/duck/Henan/S4179/2009	poultry	G8	Eurasian-2 sublineage	N6-Eurasian-2 sublineage	EA	EA	EA	EA	EA	EA(A)
A/duck/Jiangsu/S2447/2011	poultry	G8	Eurasian-2 sublineage	N6-Eurasian-2 sublineage	EA	EA	EA	EA	EA	EA(A)
A/goose/Jiangsu/S2433/2011	poultry	G8	Eurasian-2 sublineage	N6-Eurasian-2 sublineage	EA	EA	EA	EA	EA	EA(A)
A/duck/Hunan/S11090/2012	poultry	G8	Eurasian-2 sublineage	N6-Eurasian-2 sublineage	EA	EA	EA	EA	EA	EA(A)
A/duck/Hunan/S11200/2012	poultry	G8	Eurasian-2 sublineage	N6-Eurasian-2 sublineage	EA	EA	EA	EA	EA	EA(A)
A/duck/Anhui/S2193/2012	poultry	G8	Eurasian-2 sublineage	N6-Eurasian-2 sublineage	EA	EA	EA	EA	EA	EA(A)
A/duck/Guangxi/S2090/2012	poultry	G8	Eurasian-2 sublineage	N6-Eurasian-2 sublineage	EA	EA	EA	EA	EA	EA(A)
A/duck/Guangxi/149D24/2013	poultry	G8	Eurasian-2 sublineage	N6-Eurasian-2 sublineage	EA	EA	EA	EA	EA	EA(A)
A/spot-billed duck/Shanghai/SH12/2014	wild birds	G8	Eurasian-2 sublineage	N6-Eurasian-2 sublineage	EA	EA	EA	EA	EA	EA(A)
A/chicken/Shandong/36/2016	poultry	G8	Eurasian-2 sublineage	N6-Eurasian-2 sublineage	EA	EA	EA	EA	EA	EA(A)
A/duck/Shanghai/44-2/2009	poultry	G9	Eurasian-2 sublineage	N6-Eurasian-2 sublineage	ZJ-5	EA	ZJ-5	ZJ-5	EA	EA(A)
A/duck/Shanghai/46-2/2009	poultry	G9	Eurasian-2 sublineage	N6-Eurasian-2 sublineage	ZJ-5	EA	ZJ-5	ZJ-5	EA	EA(A)
A/duck/Shanghai/67-2/2009	poultry	G9	Eurasian-2 sublineage	N6-Eurasian-2 sublineage	ZJ-5	EA	ZJ-5	ZJ-5	EA	EA(A)
A/duck/Shanghai/408-1/2009	poultry	G10	Eurasian-2 sublineage	N6-Eurasian-2 sublineage	ZJ-5	ZJ-5	EA	ZJ-5	ZJ-5	EA(A)
A/duck/Shanghai/420-2/2009	poultry	G10	Eurasian-2 sublineage	N6-Eurasian-2 sublineage	ZJ-5	ZJ-5	EA	ZJ-5	ZJ-5	EA(A)
A/duck/Zhejiang/D15/2013	poultry	G10	Eurasian-2 sublineage	N6-Eurasian-2 sublineage	ZJ-5	ZJ-5	EA	ZJ-5	ZJ-5	EA(A)
A/duck/Guangxi/S1107/2010	poultry	G11	Eurasian-2 sublineage	N6-Eurasian-2 sublineage	EA	EA	EA	EA	EA	EA(B)
A/duck/Guangxi/S1211/2010	poultry	G11	Eurasian-2 sublineage	N6-Eurasian-2 sublineage	EA	EA	EA	EA	EA	EA(B)
A/duck/Guizhou/S1167/2010	poultry	G11	Eurasian-2 sublineage	N6-Eurasian-2 sublineage	EA	EA	EA	EA	EA	EA(B)

	A/duck/Hunan/S1166/2010	poultry	G11	Eurasian-2 sublineage	N6-Eurasian-2 sublineage	EA	EA	EA	EA	EA	EA(B)
	A/duck/Yunnan/YN-E2/2011	wild birds	G11	Eurasian-2 sublineage	N6-Eurasian-2 sublineage	EA	EA	EA	EA	EA	EA(B)
	A/duck/Guangxi/S4312/2011	poultry	G11	Eurasian-2 sublineage	N6-Eurasian-2 sublineage	EA	EA	EA	EA	EA	EA(B)
	A/duck/Zhejiang/S2088/2011	poultry	G12	Eurasian-2 sublineage	N6-Eurasian-2 sublineage	ZJ-5	EA	ZJ-5	ZJ-5	ZJ-5	EA(A)
	A/duck/Zhejiang/409/2013	poultry	G12	Eurasian-2 sublineage	N6-Eurasian-2 sublineage	ZJ-5	EA	ZJ-5	ZJ-5	ZJ-5	EA(A)
	A/duck/Zhejiang/413/2013	poultry	G12	Eurasian-2 sublineage	N6-Eurasian-2 sublineage	ZJ-5	EA	ZJ-5	ZJ-5	ZJ-5	EA(A)
	A/mallard/Beijing/21/2011	wild birds	G13	Eurasian-2 sublineage	N6-Eurasian-1 sublineage	EA	EA	EA	EA	EA	EA(B)
	A/Environment/Hunan/22552/2019	poultry-related environment	G13	Eurasian-2 sublineage	N6-Eurasian-1 sublineage	EA	EA	EA	EA	EA	EA(B)
	A/Environment/Chongqing/19499/2021	poultry-related environment	G13	Eurasian-2 sublineage	N6-Eurasian-1 sublineage	EA	EA	EA	EA	EA	EA(B)
	A/duck/China/J1/2012	poultry	G14	Eurasian-2 sublineage	N6-Eurasian-2 sublineage	EA	NA	EA	EA	EA	EA(A)
	A/duck/Fujian/S2169/2012	poultry	G15	Eurasian-2 sublineage	N6-Eurasian-2 sublineage	ZJ-5	ZJ-5	ZJ-5	ZJ-5	ZJ-5	EA(A)
	A/duck/Zhejiang/D2-1/2013	poultry	G15	Eurasian-2 sublineage	N6-Eurasian-2 sublineage	ZJ-5	ZJ-5	ZJ-5	ZJ-5	ZJ-5	EA(A)
	A/duck/Zhejiang/S2235/2012	poultry	G16	Eurasian-2 sublineage	N6-Eurasian-1 sublineage	ZJ-5	ZJ-5	ZJ-5	ZJ-5	ZJ-5	EA(A)
	A/duck/Zhejiang/D9/2013	poultry	G17	Eurasian-2 sublineage	N6-Eurasian-2 sublineage	ZJ-5	ZJ-5	EA	EA	ZJ-5	EA(A)
	A/duck/Zhejiang/D14/2013	poultry	G18	Eurasian-2 sublineage	N6-Eurasian-2 sublineage	ZJ-5	EA	EA	ZJ-5	ZJ-5	EA(A)
	A/spot-billed duck/Shanghai/SH148/2014	wild birds	G19	Eurasian-2 sublineage	N6-Eurasian-2 sublineage	EA	EA	EA	EA	ZJ-5	EA(A)
	A/Duck/Jiangxi/24831/2014	poultry	G20	Eurasian-2 sublineage	N6-Eurasian-2 sublineage	EA	ZJ-5	ZJ-5	EA	ZJ-5	EA(A)
	A/duck/Sichuan/04.08 CDLQ033-O/2015	poultry	G21	Eurasian-2 sublineage	N6-Eurasian-1 sublineage	EA	EA	EA	EA	NA	EA(A)
	A/duck/Ganzhou/GZ5/2015	poultry	G22	Eurasian-2 sublineage	N6-Eurasian-1 sublineage	EA	EA	ZJ-5	EA	EA	H5Nx
	A/Environment/Chongqing/33933/2018	poultry-related environment	G23	Eurasian-2 sublineage	N6-Eurasian-1 sublineage	EA	EA	ZJ-5	EA	EA	EA(B)
	A/Environment/Sichuan/39514/2019	poultry-related environment	G24	Eurasian-2 sublineage	N6-Eurasian-1 sublineage	EA	EA	EA	EA	ZJ-5	EA(A)
**H4N8**	A/chicken/Guangdong/S1010/2010	poultry	G1	Eurasian-1 sublineage	N8-North American lineage	EA	EA	EA	EA	EA	EA(A)
	A/duck/Nanjing/1102/2010	poultry	G2	Eurasian-2 sublineage	N8-North American lineage	EA	EA	EA	EA	EA	EA(A)
	A/duck/Hubei/S2114/2012	poultry	G2	Eurasian-2 sublineage	N8-North American lineage	EA	EA	EA	EA	EA	EA(A)

A/goose/Hunan/S2466/2011	poultry	G3	Eurasian-1 sublineage	N8-Eurasian lineage	EA	EA	EA	EA	EA	EA(A)
A/Mallard/Hubei/chenhu VI109/2015	wild birds	G3	Eurasian-1 sublineage	N8-Eurasian lineage	EA	EA	EA	EA	EA	EA(A)
A/duck/Chongqing/S2086/2012	poultry	G4	Eurasian-2 sublineage	N8-Eurasian lineage	EA	EA	EA	EA	EA	EA(A)
A/greylag goose/Changsha/CS-1983/2013	wild birds	G5	Eurasian-1 sublineage	N8-North American lineage	EA	EA	EA	ZJ-5	EA	EA(A)
A/greylag goose/Changsha/CS-510/2013	wild birds	G6	Eurasian-1 sublineage	N8-North American lineage	H9N2	EA	EA	ZJ-5	EA	EA(A)
A/Environment/Guangdong/77233/2014	poultry-related environment	G7	Eurasian-1 sublineage	N8-North American lineage	EA	EA	ZJ-5	EA	ZJ-5	EA(B)
A/duck/Guangdong/DGQTSJ147P/2015	unknown	G8	Eurasian-1 sublineage	N8-North American lineage	EA	EA	EA	EA	ZJ-5	EA(A)
A/chicken/Jiangsu/YC/2015	poultry	G9	Eurasian-1 sublineage	N8-North American lineage	EA	ZJ-5	EA	EA	ZJ-5	EA(A)
A/Environment/Guangxi/28327/2017	poultry-related environment	G10	Eurasian-2 sublineage	N8-North American lineage	ZJ-5	EA	EA	EA	ZJ-5	EA(A)

* Abbreviation of sublineages in internal genes: EA, Eurasian wild bird gene pool; EA(A), Eurasian wild bird gene pool (allele A); EA(B), Eurasian wild bird gene pool (allele B); NA, North American wild bird gene pool; ZJ-5, ZJ-5 sublineage (gene pool); H9N2, poultry H9N2(ZJ-HJ/07); H5Nx, H5Nx sublineage.
