# Supplementary material for: Cross-Species Transmission Potential of H4 Avian Influenza Viruses in China: Epidemiological and Evolutionary Study
Source: Viruses. 2024 Feb 24;16(3):353. doi: 10.3390/v16030353 (PMC10974465; doi:10.3390/v16030353)
Supplement: Supplementary file 1 [file viruses-16-00353-s001.zip › Supplementary Table 3.pdf]

Table S3. List of H4 AIVs and genotypes with key molecular markers in China

Subtype	Genotype	isolate	HA	PB2					PB1	PA		NP		M2		
			Altered receptor specificity	Enhanced virus polymerase activity and virulence in mice	Host signature amino acids through statistical methods (avian to human)									Increased resistance to amantadine and rimantadine		
			V214I	K482R	D9N	M64T	T105M	K702R	R327K	R57Q	S409N	V33I	D455E	L26F	V27I/A	S31N
**H4N2**	G3	A/duck/Guangdong/S1469/2010	V	K	D	M	T	K	R	R	S	V	D	L	I	S
	G4	A/wild bird/China/Y13/2019	V	K	D	M	T	R	R	R	S	V	D	L	I	S
	G4	A/duck/Hunan/S11313/2012	V	R	D	M	T	K	R	R	S	V	D	L	V	S
	G9	A/duck/Jiangsu/1-15/2011	V	K	D	M	A	K	R	R	S	V	D	L	V	N
	G10	A/duck/Hubei/S2213/2012	V	K	D	M	T	K	R	R	S	V	D	L	I	S
	G14	A/duck/Hubei/S2227/2012	V	K	D	M	T	K	R	R	N	V	D	L	I	S
	G15	A/duck/Hunan/01.16 YYGK227-P/2014	V	K	D	M	T	K	K	R	S	V	D	L	V	S
	G16	A/Environment/Hunan/28094/2014	V	K	D	M	T	K	R	R	S	V	D	L	I	S
	G17	A/duck/Zhejiang/925170/2014	V	K	D	M	T	K	R	R	N	V	D	L	V	S
	G17	A/duck/Zhejiang/925161/2014	V	K	D	M	T	K	R	R	N	V	D	L	V	S
	G18	A/duck/Zhejiang/727145/2014	V	K	D	M	T	K	R	R	N	V	D	L	V	S
	G18	A/duck/Zhejiang/77140/2014	V	K	D	M	T	K	R	R	N	V	D	L	V	S
	G18	A/duck/Zhejiang/77127/2014	V	K	D	M	T	K	R	R	N	V	D	L	V	S
	G20	A/Environment/Guangdong/34254/2019	V	K	D	M	T	K	R	R	S	V	D	L	A	S
	G22	A/Environment/Guangxi/09909/2021	V	K	D	M	T	K	R	R	S	V	D	L	V	N
	G23	A/Environment/Guangdong/34255/2019	V	K	D	M	T	K	R	R	S	V	D	L	A	S
**H4N6**	G2	A/mallard/Yan chen/2005	V	K	D	M	T	R	R	R	S	V	D	L	V	S
	G3	A/mallard/ZhaLong/88/2004	V	K	D	I	A	K	R	R	S	V	D	L	V	S
	G4	A/duck/Shanghai/Y20/2006	V	K	D	M	M	K	R	R	S	V	D	L	V	S
	G5	A/duck/Hunan/S1012/2009	V	K	D	M	T	K	R	R	S	V	E	L	V	S
	G6	A/duck/Shanghai/421-2/2009	I	K	D	M	T	K	R	R	S	V	D	L	V	S
	G7	A/Environment/Hubei/02/2009	V	K	D	M	T	K	K	R	S	V	D	L	V	S
	G7	A/duck/Yunnan/YN-1/2011	V	K	D	M	T	K	R	Q	S	V	D	L	V	S
	G7	A/Environment/Hunan/00600/2020	V	K	D	M	T	R	R	R	S	V	D	L	V	S
	G8	A/weiyangshui/Jiangxi/14/2009	V	K	D	M	T	K	R	R	S	V	D	L	I	S
	G8	A/duck/Guangxi/S2090/2012	V	K	D	M	M	K	R	R	S	V	D	L	V	S
	G8	A/duck/Anhui/S4155/2009	V	K	D	M	T	K	R	R	S	I	D	L	V	S

	G8	A/duck/Anhui/S2193/2012	V	K	D	M	T	K	R	R	S	V	D	L	V	N
	G8	A/duck/Guangxi/149D24/2013	V	K	D	M	T	K	K	R	S	V	D	L	V	S
	G9	A/duck/Shanghai/67-2/2009	V	K	D	M	T	K	R	R	S	V	D	L	I	S
	G9	A/duck/Shanghai/46-2/2009	V	K	D	M	T	K	R	R	S	V	D	L	I	S
	G9	A/duck/Shanghai/44-2/2009	V	K	D	M	T	K	R	R	S	V	D	L	I	S
	G15	A/duck/Zhejiang/D2-1/2013	V	K	D	M	A	K	R	R	S	V	D	L	V	S
	G23	A/Environment/Chongqing/33933/2018	V	K	D	M	T	K	R	Q	S	V	D	L	V	S
	G24	A/Environment/Sichuan/39514/2019	V	K	D	T	T	K	R	R	S	V	D	L	V	S
**H4N8**	G1	A/chicken/Guangdong/S1010/2010	V	K	D	M	T	K	R	R	S	V	D	F	I	S
	G3	A/Mallard/Hubei/chenhu VI109/2015	V	K	D	M	T	K	R	R	S	I	D	L	V	S
	G3	A/goose/Hunan/S2466/2011	V	K	N	M	T	K	R	R	S	V	D	L	V	S
	G5	A/greylag goose/Changsha/CS-1983/2013	V	K	D	M	T	K	R	R	S	V	D	L	I	S
	G6	A/greylag goose/Changsha/CS-510/2013	V	K	D	M	T	K	R	R	S	V	D	L	I	S
	G9	A/chicken/Jiangsu/YC/2015	V	K	D	M	T	K	R	R	S	V	D	L	A	S
