# Supplementary material for: Cross-Species Transmission Potential of H4 Avian Influenza Viruses in China: Epidemiological and Evolutionary Study
Source: Viruses. 2024 Feb 24;16(3):353. doi: 10.3390/v16030353 (PMC10974465; doi:10.3390/v16030353)
Supplement: Supplementary file 1 [file viruses-16-00353-s001.zip › Supplementary Table 4.pdf]

Table S4. Potential glycosylation motifs of HA protein.

isolate name	2 NYT	18 NGT	162 NLT	294 NIS	438 NDS	481 NGT
A/wild bird/China/Y13/2019_H4N2	NYT	NGT	NLT	NIS		NGT
A/Environment/Hunan/12618/2017_H4N2	NYT	NGT	NLT	NIS		NGT
A/Environment/Guangxi/09909/2021_H4N2	NYT	NGT	NLT	NIS		NGT
A/Environment/Chongqing/19499/2021_H4N6	NYT	NGT	NLT	NIS		NGT
A/Environment/Fujian/23044/2018_H4N6	NYT	NGT	NLT	NIS		NGT
A/weiyangshui/Jiangxi/14/2009_H4N6	NYT	NGT	NLT	NIS		NGT
A/jilongshizi/Jiangxi/19/2009_H4N6	NYT	NGT	NLT	NIS		NGT
A/duck/Yunnan/YN-E2/2011_H4N6	NYT	NGT	NLT	NIS		NGT
A/duck/Hunan/S2046/2011_H4N2	NYT	NGT	NLT	NIS		NGT
A/duck/Henan/S4179/2009_H4N6	NYT	NGT	NLT	NIS		NGT
A/duck/Guizhou/S1167/2010_H4N6	NYT	NGT	NLT	NIS		NGT
A/duck/Guangxi/S1107/2010_H4N6	NYT	NGT	NLT	NIS		NGT
A/duck/Guangxi/S1211/2010_H4N6	NYT	NGT	NLT	NIS		NGT
A/duck/Hunan/S1166/2010_H4N6	NYT	NGT	NLT	NIS		NGT
A/spot-billed duck/Shanghai/SH148/2014_H4N6	NYT	NGT	NLT	NIS		NGT
A/duck/Hunan/S11200/2012_H4N6	NYT	NGT	NLT	NIS		NGT
A/duck/Hunan/S11090/2012_H4N6	NYT	NGT	NLT	NIS		NGT
A/duck/Zhejiang/D14/2013_H4N6	NYT	NGT	NLT	NIS		NGT
A/duck/Guangxi/S2090/2012_H4N6	NYT	NGT	NLT	NIS		NGT
A/duck/Fujian/S2169/2012_H4N6	NYT	NGT	NLT	NIS		NGT
A/duck/Guangxi/S4312/2011_H4N6	NYT	NGT	NLT	NIS		NGT
A/duck/China/J1/2012_H4N6	NYT	NGT	NLT	NIS		NGT
A/chicken/Shandong/36/2016_H4N6	NYT	NGT	NLT	NIS		NGT
A/duck/Zhejiang/D2-1/2013_H4N6	NYT	NGT	NLT	NIS		NGT
A/duck/Shanghai/67-2/2009_H4N6	NYT	NGT	NLT	NIS		NGT
A/duck/Shanghai/46-2/2009_H4N6	NYT	NGT	NLT	NIS		NGT
A/duck/Shanghai/44-2/2009_H4N6	NYT	NGT	NLT	NIS		NGT
A/duck/Shanghai/420-2/2009_H4N6	NYT	NGT	NLT	NIS		NGT
A/duck/Shanghai/408-1/2009_H4N6	NYT	NGT	NLT	NIS		NGT
A/duck/Anhui/S4155/2009_H4N6	NYT	NGT	NLT	NIS		NGT
A/duck/Jiangxi/S21055/2012_H4N2	NYT	NGT	NLT	NIS		NGT
A/duck/Jiangxi/S21046/2012_H4N2	NYT	NGT	NLT	NIS		NGT
A/duck/Chongqing/S2086/2012_H4N8	NYT	NGT	NLT	NIS		NGT
A/duck/Anhui/S2193/2012_H4N6	NYT	NGT	NLT	NIS		NGT
A/chicken/Shandong/S2510/2012_H4N2	NYT	NGT	NLT	NIS		NGT
A/duck/Zhejiang/S2088/2011_H4N6	NYT	NGT	NLT	NIS		NGT
A/duck/Zhejiang/413/2013_H4N6	NYT	NGT	NLT	NIS		NGT
A/duck/Zhejiang/409/2013_H4N6	NYT	NGT	NLT	NIS		NGT
A/duck/Hunan/8-19/2009_H4N2	NYT	NGT	NLT	NIS		NGT
A/Environment/Sichuan/39514/2019_H4N6	NYT	NGT	NLT	NIS		NGT
A/chicken/Guangxi/04.10 NM140-O/2015_H4N3	NYT		NLT	NIS		NGT
A/Environment/Guangxi/28327/2017_H4N8	NYT	NGT	NLT	NIS		NGT
A/duck/Jiangsu/1-15/2011_H4N2	NYT	NGT	NLT	NIS		NGT

A/duck/Guangxi/149D24/2013_H4N6	NYT	NGT	NLT	NIS	NGT
A/Environment/Hunan/28094/2014_H4N2	NYT	NGT	NLT	NIS	NGT
A/duck/Zhejiang/S2235/2012_H4N6	NYT	NGT	NLT	NIS	NGT
A/duck/Guangxi/125D17/2012_H4N2	NYT	NGT	NLT	NIS	NGT
A/duck/Shanghai/29-1/2009_H4N2	NYT	NGT	NLT	NIS	NGT
A/spot-billed duck/Shanghai/SH12/2014_H4N6	NYT	NGT	NLT	NIS	NGT
A/mallard/Beijing/21/2011_H4N6	NYT	NGT	NLT	NIS	NGT
A/duck/Jiangxi/S2443/2012_H4N6	NYT	NGT	NLT	NIS	NGT
A/duck/Hunan/S11893/2012_H4N6	NYT	NGT	NLT	NIS	NGT
A/duck/Ganzhou/GZ5/2015_H4N6	NYT	NGT	NLT	NIS	NGT
A/Environment/Fujian/46611/2016_H4N2	NYT	NGT	NLT	NIS	NGT
A/Environment/Fujian/46605/2016_H4N2	NYT	NGT	NLT	NIS	NGT
A/Duck/Jiangxi/24831/2014_H4N6	NYT	NGT	NLT	NIS	NGT
A/duck/Zhejiang/D9/2013_H4N6	NYT	NGT	NLT	NIS	NGT
A/duck/Zhejiang/D15/2013_H4N6	NYT	NGT	NLT	NIS	NGT
A/duck/Zhejiang/925170/2014_H4N2	NYT	NGT	NLT	NIS	NGT
A/duck/Zhejiang/925161/2014_H4N2	NYT	NGT	NLT	NIS	NGT
A/duck/Zhejiang/727145/2014_H4N2	NYT	NGT	NLT	NIS	NGT
A/duck/Hubei/S2114/2012_H4N8	NYT	NGT	NLT	NIS	NGT
A/Environment/Hubei/02/2009_H4N6	NYT	NGT	NLT	NIS	NGT
A/duck/Henan/S1091/2010_H4N6	NYT	NGT	NLT	NIS	NGT
A/chicken/Hunan/S1267/2010_H4N6	NYT	NGT	NLT	NIS	NGT
A/chicken/Hunan/S1248/2010_H4N6	NYT	NGT	NLT	NIS	NGT
A/duck/Yunnan/YN-1/2011_H4N6	NYT	NGT	NLT	NIS	NGT
A/duck/Sichuan/S4202/2011_H4N2	NYT	NGT	NLT	NIS	NGT
A/duck/Nanjing/1102/2010_H4N8	NYT	NGT	NLT	NIS	NGT
A/duck/Hunan/S11313/2012_H4N2	NYT	NGT	NLT	NIS	NGT
A/duck/Sichuan/04.08 CDLQ169-O/2015_H4N2	NYT	NGT	NLT	NIS	NGT
A/duck/Sichuan/04.08 CDLQ145-P/2015_H4N2	NYT	NGT	NLT	NIS	NGT
A/duck/Sichuan/04.08 CDLQ146-O/2015_H4N2	NYT	NGT	NLT	NIS	NGT
A/Environment/Chongqing/45438/2014_H4N2	NYT	NGT	NLT	NIS	NGT
A/duck/Zhejiang/77140/2014_H4N2		NGT	NLT	NIS	NGT
A/duck/Zhejiang/77127/2014_H4N2		NGT	NLT	NIS	NGT
A/mallard/Beijing/16/2016_H4N6	NYT	NGT	NLT	NIS	NGT
A/mallard/Beijing/10/2016_H4N6	NYT	NGT	NLT	NIS	NGT
A/Environment/Hunan/22552/2019_H4N6	NYT	NGT	NLT	NIS	NGT
A/Environment/Chongqing/33933/2018_H4N6	NYT	NGT	NLT	NIS	NGT
A/Environment/Guangxi/13283/2019_H4N2	not available	NGT	NLT	NIS	NGT
A/Environment/Guangdong/34241/2019_H4N2	NYT	NGT	NLT	NIS	NGT
A/Environment/Hunan/00600/2020_H4N6	NYT	NGT	NLT	NIS	NGT
A/Environment/Guangxi/01898/2021_H4N2	NYT	NGT	NLT	NIS	NGT
A/Environment/Guangdong/14072/2020_H4N2	NYT	NGT	NLT	NIS	NGT
A/Environment/Guangxi/32049/2017_H4N6	NYT	NGT	NLT	NIS	NGT
A/Environment/Zhejiang/22264/2017_H4N2	NYT	NGT	NLT	NIS	NGT
A/Environment/Jiangxi/13772/2020_H4N6	NYT	NGT	NLT	NIS	NGT

A/Environment/Guangdong/34255/2019_H4N2	NYT	NGT	NLT	NIS		NGT
A/Environment/Guangdong/34254/2019_H4N2	NYT	NGT	NLT	NIS		NGT
A/duck/Hubei/S2227/2012_H4N2	NYT	NGT	NLT	NIS		NGT
A/duck/Hubei/S2213/2012_H4N2	NYT	NGT	NLT	NIS		NGT
A/duck/Zhejiang/925088/2014_H4N2	NYT	NGT	NLT	NIS		NGT
A/duck/Zhejiang/925028/2014_H4N2	NYT	NGT	NLT	NIS		NGT
A/duck/Sichuan/04.08 CDLQ033-O/2015_H4N6	NYT	NGT	NLT	NIS		NGT
A/duck/Sichuan/04.08 CDLQ021-P/2015_H4N3	NYT	NGT	NLT	NIS		NGT
A/duck/Sichuan/04.08 CDLQ 020-P/2015_H4N3	NYT	NGT	NLT	NIS		NGT
A/goose/Jiangsu/S2433/2011_H4N6	NYT	NGT	NLT	NIS		NGT
A/duck/Jiangsu/S2447/2011_H4N6	NYT	NGT	NLT	NIS		NGT
A/mallard/ZhaLong/88/2004_H4N6	NYT	NGT	NLT	NIS		NGT
A/duck/Shanghai/Y20/2006_H4N6	NYT	NGT	NLT	NIS	NDS	NGT
A/duck/Guangxi/912/2008_H4N2	NYT	NGT	NLT	NIS		NGT
A/mallard/Yan chen/2005_H4N6	NYT	NGT	NLT	NIS		NGT
A/duck/Shanghai/421-2/2009_H4N6	NYT	NGT	NLT	NIS		NGT
A/Mallard/Hubei/chenhu VI109/2015_H4N8	NYT	NGT	NLT	NIS		NGT
A/environment/sichuan/322076/2015_H4N6	NYT	NGT	NLT	NIS		NGT
A/environment/sichuan/322074/2015_H4N6	NYT	NGT	NLT	NIS		NGT
A/Environment/Sichuan/03392/2015_H4N2	NYT	NGT	NLT	NIS		NGT
A/Environment/Chongqing/45279/2015_H4N3	NYT	NGT	NLT	NIS		NGT
A/goose/Hunan/S2466/2011_H4N8	NYT	NGT	NLT	NIS		NGT
A/duck/Hunan/S1012/2009_H4N6	NYT	NGT	NLT	NIS		NGT
A/duck/Hunan/01.16 YYGK227-P/2014_H4N2	NYT	NGT	NLT	NIS		NGT
A/greylag goose/Changsha/CS-510/2013_H4N8	NYT	NGT	NLT	NIS		NGT
A/greylag goose/Changsha/CS-1983/2013_H4N8	NYT	NGT	NLT	NIS		NGT
A/goose/Guangdong/S1780/2012_H4N2	NYT	NGT	NLT	NIS		NGT
A/duck/Guangdong/S1123/2012_H4N2	NYT	NGT	NLT	NIS		NGT
A/duck/Guangdong/S4040/2011_H4N2	NYT	NGT	NLT	NIS		NGT
A/duck/Jiangxi/S3261/2009_H4N2	NYT	NGT	NLT	NIS		NGT
A/duck/Guangdong/S1469/2010_H4N2	NYT	NGT	NLT	NIS		NGT
A/chicken/Guangdong/S1010/2010_H4N8	NYT	NGT	NLT	NIS		NGT
A/Environment/Guangdong/77233/2014_H4N8	NYT	NGT	NLT	NIS		NGT
A/duck/Guangdong/DGQTSJ147P/2015_H4N8	NYT	NGT	NLT	NIS		NGT
A/chicken/Jiangsu/YC/2015_H4N8	NYT	NGT	NLT	NIS		NGT
A/Duck/Nanchang/4-165/2000_H4N6	NYT	NGT	NLT	NIS		NGT
A/duck/Fujian/S1487/2009_H4N3	NYT	NGT	NLT	NIS		NGT
A/duck/Hong Kong/365/1978_H4N6	NYT	NGT	NLT	NIS		NGT
A/duck/Hong Kong/24/1976_H4N2	NYT	NGT	NLT	NIS		NGT
