# Supplementary material for: Cross-Species Transmission Potential of H4 Avian Influenza Viruses in China: Epidemiological and Evolutionary Study
Source: Viruses. 2024 Feb 24;16(3):353. doi: 10.3390/v16030353 (PMC10974465; doi:10.3390/v16030353)
Supplement: Supplementary file 1 [file viruses-16-00353-s001.zip › Supplementary Table 5.pdf]

Table S5. Genotypes of H4N2, H4N3, H4N6 and H4N8 from wild birds, poultry and unknown

H4N2			
Genotype	wild birds	poultry	unknown
G1	1		
G2		2	
G3		4	
G4	1	3	
G5		1	
G6		1	
G7		3	
G8		1	
G9		1	
G10		2	
G11		1	
G12		1	
G13		1	
G14		1	
G15		1	
G16		2	
G17		2	
G18		4	
G19		2	
G20		3	
G21		2	
G22		3	
G23		1	
G24		1	

H4N6			
Genotype	wild birds	poultry	unknown
G1	1		
G2	1	1	
G3	1		
G4		1	
G5		3	
G6		1	
G7	3	10	
G8	1	12	
G9		3	
G10		3	
G11	1	5	
G12		3	
G13		1	
G14		2	
G15		1	
G16		1	
G17		1	
G18	1		
G19		1	
G20		1	
G21		1	
G22		1	
G23	1	2	
G24		1	

H4N3			
Genotype	wild birds	poultry	unknown
G1		1	
G2		1	
G3		1	
G4		2	

H4N8			
Genotype	wild birds	poultry	unknown
G1		1	
G2		2	
G3	1	1	
G4		1	
G5	1		
G6	1		
G7		1	
G8			1
G9		1	
G10		1	
